# Axon Regeneration and Functional Recovery after Spinal Cord Injury is Enhanced by Allele-Specific ApoE Neuronal Action through LRP8

**DOI:** 10.1101/2025.10.10.681747

**Published:** 2025-10-13

**Authors:** Ramakrishnan Kannan, Xingxing Wang, LaShae Nicholson, Nancy Z. Lin, Elisa M. Howard, Atrayee Basu, Ines Ingabire, Yuichi Sekine, Stephen M. Strittmatter

**Affiliations:** 1 Departments of Neuroscience and Neurology, Program in Cellular Neuroscience, Neurodegeneration and Repair, Yale School of Medicine, 100 College Street, New Haven, CT 06510 USA

## Abstract

Adult CNS trauma frequently causes neuronal disconnection and persistent deficits due to failed axon regeneration. While model system screening has identified multiple candidate neural repair pathways, ApoE–LRP8 signaling is unique in being implicated clinically. Here, we show that cortical axon regeneration requires LRP8 and is modified by *APOE* variants. ApoE2-expressing mice show reparative corticospinal and raphespinal axon growth with greater motor function than controls after spinal cord injury. Distinct from ApoE in other settings, there is no change in inflammation or scarring. After axotomy, ApoE exerts allele-specific effects on LRP8 localization and signaling in cortical neurons. *APOE* alleles regulate synaptic organization gene expression by cortical neurons after injury, with little effect on glial gene expression. AAV-mediated overexpression of ApoE2 in mice after spinal trauma increases locomotor recovery and reparative axon growth. Thus, ApoE–LRP8 signaling for axon regrowth following CNS trauma provides a potential therapeutic intervention site.

## INTRODUCTION

Many acute injuries to the central nervous system interrupt axonal connectivity. This is most dramatic in traumatic spinal cord injury (SCI) where very few neurons are lost but communication between the brain and distal spinal cord are nil after trauma, and neurological deficits can be pronounced(1). Unfortunately, repair of connectivity through axonal regeneration is extremely limited in adult mammals so recovery is typically poor(2). While overcoming limitations to reparative axon growth provides a possible avenue towards clinical improvement, the 300,000 individuals in the United States with persistent neurologic deficits after SCI have no therapeutic option today(3).

A range of non-clinical experiments have sought to define molecular restraints upon axonal growth and plasticity after injury(4, 5). Potential molecular approaches to overcome the brakes on neural repair have not yet entered clinical practice, though several promising approaches are now being tested in clinical trials(6, 7). This includes blockade of myelin-derived inhibitors and interruption of chondroitin sulfate proteoglycan action. Training and electrical stimulation have been used to coax neurons into a more plastic state to support functional recovery and have achieved early clinical promise(8, 9). Despite these advancements there remains no therapeutic intervention to improve recovery after traumatic spinal cord injury.

We conducted a genome wide loss of function screen in cortical neurons to identify those genes modifying the extent of axonal regeneration *in vitro*(10). Over 500 genes with significant effects were identified and dozens were validated with an *in vivo* optic nerve regeneration screen(11). Rab27B(10), rabphilin-3A(12) and interleukin-33(11) were further confirmed using gene knockout mice in spinal cord injury or optic nerve crush experiments. In addition, specific experiments have shown that PTEN(13), SOCS3(14), KLF4(15), neurotrophins, and several transcription factors titrate the regenerative capacity of CNS axons(16–18). Amongst these numerous candidates for enhancing neural repair, evidence for contribution to clinical recovery via genetic association would be extremely helpful to validate and assess relevance.

Genome wide association studies to seek variants that might modulate the neural repair process have not been reported for SCI, but studies of traumatic brain injury (TBI) have been completed. The ApoE4 variant reached genome-wide significance for TBI outcome(19), and has been linked to SCI recovery clinically and experimentally(20–23). In our screen(10), loss of the ApoE receptor LRP8 suppressed axon regeneration. Here, we examined whether an ApoE–LRP8 signaling axis might regulate the extent of neural repair after spinal cord injury. We report that ApoE has an allele-specific effect on an LRP8-dependent axonal regeneration pathway. ApoE2 knock-in mice show selective changes of neuronal gene expression, enhanced axon regeneration and greater functional recovery after traumatic spinal cord injury. Furthermore, increasing ApoE2 after trauma enhances neural repair and recovery. Notably, the neuronal selectivity of ApoE–LRP8 action in SCI recovery differs from ApoE action on protein clearance, transport, cell metabolism and inflammation in neurodegenerative studies, indicating the diverse action of ApoE in multiple neurological conditions.

## RESULTS

### ApoE ligand and neuronal LRP8 receptor are indispensable for axon growth

**Fig. 1.**
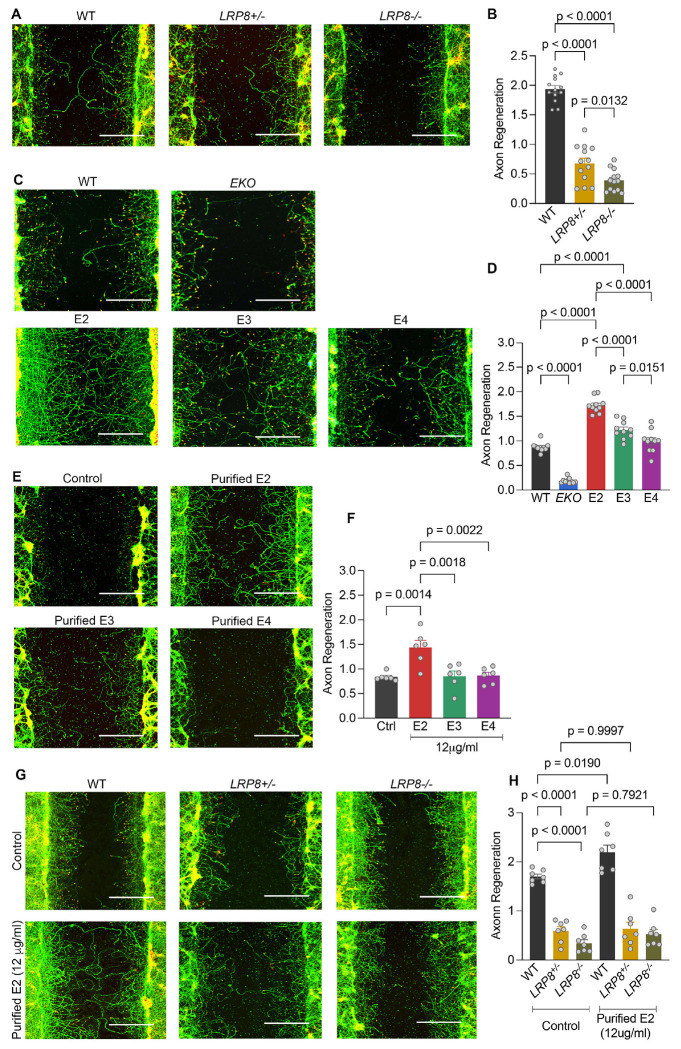
E2 promotes axon regeneration through neuronal LRP8 receptor Axotomized primary cortical neuron cultures stained with ßIII-tubulin (green) and phalloidin (red) at 15 days after plating and 7 days after axotomy. A portion of regeneration zone is shown here. For each genotype, datapoints are an average of 60 wells from independent biological replicates. Regeneration scores are normalized to WT. Scale bars, 200 μm. (A) Photomicrographs of axon regeneration from WT, *LRP8*^+/−^ and *LRP8*^−/−^ cortical neuron cultures. (B) Axon regeneration index for (A) from 13 mice for each group. (C) Representative immunofluorescence micrographs of axon regeneration from WT, EKO, E2, E3 and E4 cortical neuron cultures. (D) Axon regeneration index for (C) from 10 mice for each group. (E) Photomicrographs of axon regeneration from EKO cortical neurons treated with purified ApoE lipoprotein particles (12 μg/ml) from astrocytic conditioned media (ACM). (F) Axon regeneration index for (E) from 6 mice for each group. (G) Photomicrographs of axon regeneration from WT, *LRP8*^−/+^ and *LRP8*^−/−^ cortical neuron cultures treated with purified E2 lipoparticles with equal amount of PBS as control. (H) Axon regeneration index for (E) from 7 mice for each group. Data presented as mean ± SEM. One-way ANOVA with Tukey’s multiple comparisons for (B): F = 145.2, dF = 2; (D): F = 127.4, dF = 4; (F): F = 9.3, dF = 3; (H): F = 54.5, dF = 5.

Our previous genome-wide shRNA screen identified dozens of genes for which loss of function modified the success of axon regeneration *in vitro* as well as *in vivo* after optic nerve crush and spinal cord trauma(10–12). Within the Low-density lipoprotein Receptor-related Protein (LRP) family, suppression of *Lrp6* and *Lrp8* expression significantly reduced axon regeneration (Fig. S1A). LRP8 is known to be enriched in neurons and to modulate brain development and adult synaptic plasticity(24–27). Consistent with the shRNA data, deletion of mouse *Lrp8* (Fig. S1B) inhibits axon regeneration from cortical neurons relative to wild-type in a gene dosage-dependent manner (Fig. 1A and B).

ApoE and Reelin are known physiological ligands for LRP8 receptor in the brain(28), where ApoE is a major lipid and cholesterol transporter. However, the role of ApoE isoforms in CNS regeneration has yet to be fully elucidated. We considered whether ApoE isoforms have varying effects on regenerative axon growth via LRP8. Axon regeneration after mechanical trauma was assessed for cortical neurons cultured from mice homozygous for knock-in (KI) of the three common human *APOE* alleles (E2, E3 or E4) or from *ApoE* null mice (EKO) or from wild-type mice. The E2 cortical neuron cultures display a significant increase in axon regeneration, while the EKO neurons exhibit reduced regeneration (Fig. 1C and D). Taken together, these data show that ApoE and its receptor LRP8 are crucial for axon regeneration with expression of ApoE2 stimulating growth.

### Secreted lipidated E2 modulates LRP8 signaling for reparative axon growth

Astrocytes are known to be the primary source of secreted lipidated ApoE in the brain(29). We sought to evaluate the role of astrocyte-secreted ApoE in axon regeneration. Cortical neuron cultures utilized for axon regeneration assays *in vitro* contain nearly 90% neurons, with astrocytes being the most prevalent glial cell(10). As a first step, we assessed cell type specific differences in expression from human *APOE* knock-in alleles by immunoblotting tissue homogenates as well as cellular and secreted fractions of separately cultured cortical neurons and astrocytes. In cortical tissue, the levels of ApoE were uniform across the three different *APOE* knock-in mice (Fig. S1C). The expression and secretion of E2 was similar between the different fractions (Fig. S1D). The level of E4 was reduced in cellular and secreted neuronal extracts as compared to E3 (Fig. S1E), but was comparable for astrocyte cultures (Fig. S1F).

To test the role of secreted ApoE in axon regeneration, we used astrocyte-conditioned medium from the three humanized ApoE-KI models together with EKO neuron cultures, thereby eliminating any influence of neuronal ApoE or wild-type mouse ApoE. Cultures of EKO astrocytes yielded control ApoE-free medium. Astrocyte-conditioned medium extracts from E2 and E3 facilitated regeneration of EKO neurons more effectively than does E4 medium (Fig. S1G and S1H). To exclude the possibility of differential growth factor levels in astrocyte-conditioned medium and to establish the primary role of lipidated secreted human ApoE in regeneration, we immuno-purified ApoE from astrocyte-conditioned medium. Native gel electrophoresis of purified fractions revealed predominant association of ApoE isoforms with high molecular weight lipid-rich particles (Fig. S1I).

Purified E2 lipoprotein particles substantially increased axon regeneration of EKO neurons compared to E3 and E4 (Fig. 1E and 1F). To determine whether E2 lipoparticles signal through neuronal LRP8 receptor, we conducted an *in vitro* epistasis experiment with wild-type and LRP8 null neurons. Secreted E2 significantly enhanced axon regeneration of wild-type but not *Lrp8*^−/−^ and *Lrp8*^−/+^ cortical neurons indicating that E2 modulates axon regeneration through neuronal Lrp8 signaling (Fig. 1G and H).

### E2 mice show enhanced CNS regeneration with improved functional recovery from SCI

**Fig. 2.**
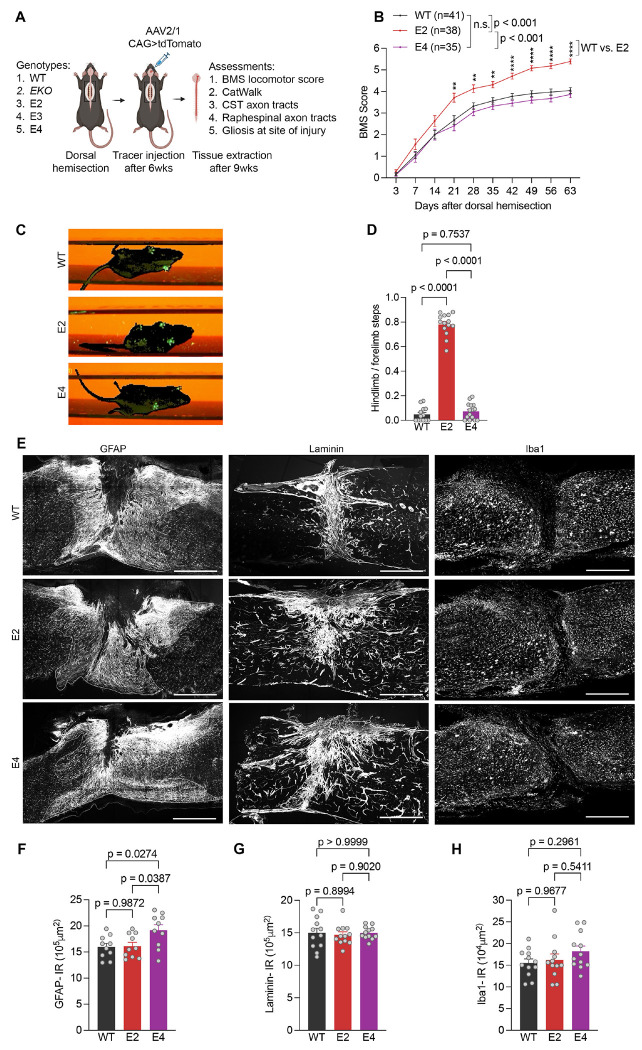
Enhanced locomotor recovery in E2 mice after dorsal thoracic over-hemisection. (A) Experimental overview to evaluate functional and anatomical features of motor recovery in SCI mice with different ApoE-KI alleles. (B) BMS open-field locomotion assessment for WT (*n* = 42), E2 (*n* = 38) and E4 (*n* = 35) SCI mice. Performance was scored for each animal every consecutive week. Data as mean ± SEM and *p* values calculated by repeated measure multi-point ANOVA across time series followed by post hoc Tukey’s multiple comparisons test between genotypes at indicated time points. ****p<0.0001, **p<0.01 (C) Single frame of CatWalk video showing fore and hindlimb paw footprints in green for WT, E2 and E4 mice 9 weeks after SCI. (D) Limb coordination index of WT (*n* = 13), E2 (*n* = 14) and E4 (*n* = 15) mice, 9 weeks after SCI. (E) Photomicrographs of sagittal spinal cord sections at thoracic SCI epicenter. Inflammation was assessed by quantitating GFAP, Laminin and Iba1 immunoreactive area at the injury site for *n* = 10 WT, E2 and E4 mice. Scale bar, 500 μm. (F) Quantification of GFAP immunoreactive area at injury site. (G) Quantification of Laminin immunoreactive area at injury site. (H) Quantification of Iba1 immunoreactive area at injury site. Data shown as mean ± SEM and *p* values calculated by one-way ANOVA with Tukey’s multiple comparisons test for (D): F = 455.1, dF = 2; (F): F = 4.77, dF = 2; (G): F = 0.13, dF = 2; (H): F = 1.45, dF = 2.

We hypothesized that the effect of ApoE variants on recovery from clinical TBI and SCI relates to titration of reparative axonal growth through neuronal LRP8, and sought to test this for mouse SCI. Before investigating anatomical and behavioral SCI recovery, we confirmed that the development of two relevant descending spinal tracts was normal without injury. Both corticospinal (CST) and raphespinal projections show normal anatomy in the three ApoE-KI strains and in ApoE null mice (Fig. S2A and S2B). Therefore, to assess the role of ApoE alleles in CNS repair and functional recovery *in vivo*, we performed thoracic spinal cord dorsal over-hemisection in adult ApoE-KI mice (Fig. 2A). By Basso Mouse Score (BMS) locomotor evaluation in the open field, all experimental groups had normal hindlimb function before injury, but essentially complete paralysis 3 days after injury. We tracked locomotor ability of injured mice until 63 days post-SCI (Fig. 2B). From 21 days onwards, recovery of the E2 group was significantly greater than for WT or E4 mice (Fig. 2B). Nearly all E2 mice achieved extensive ankle movement and hindlimb support while most WT and E4 mice exhibited limited ankle movement and absent hindlimb weight support. Locomotor recovery in E3 mice was indistinguishable from WT mice, and the EKO mice had less restoration of hindlimb function after injury (Fig. S2C). In addition, we assessed interlimb coordination during locomotion through a linear track by CatWalk analysis at 8-wks post-SCI (Fig. 2C). Consistently, E2 mice displayed better limb coordination and balance with paw support compared to WT and E4 mice (Fig. 2D). The automated analysis of paw prints detected hindlimb steps for more than 75% of forelimb steps in E2 mice but fewer than 5% of forelimb steps of WT and E4 mice. Thus, ApoE expression has a prominent role in SCI recovery with ApoE2 being most beneficial for locomotor outcome, and with ApoE3, ApoE4 and mouse ApoE each supporting more improvement than the ApoE null state.

After SCI, reactive astrocytes, microglia, peripheral immune cells and fibroblasts invade the SCI lesion core and alter tissue damage during the acute and subacute phases. Therefore, ApoE-regulated locomotor recovery might be attributable to either differential neuroprotection or reparative axonal growth, though the late behavioral recovery favors a neural repair mechanism rather than neuroprotection. As assessed by GFAP-positive astrocytosis, fibroblast-derived laminin deposition or Iba1-positive microglial presence, there was no difference between the spinal cord lesion sites of E2, E4 and WT mice (Fig. 2E–2H). This further supports the hypothesis that axonal regrowth rather than neuroprotection accounts for improved BMS after SCI.

**Fig. 3.**
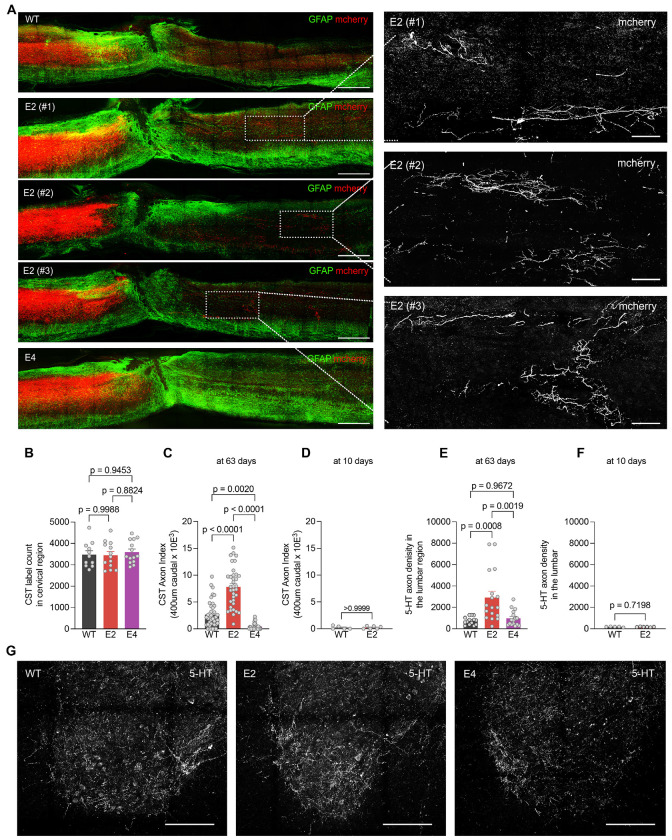
Enhanced regeneration of corticospinal and raphespinal axon tracts in E2 mice after SCI. (A) Sagittal photomicrographs of spinal cord spanning the thoracic dorsal over-hemisection lesion at 63 days after injury. AAV-mCherry anatomical tracer used to label regenerating CST axons (red), and GFAP immunohistology for reactive astrocytes (green). Dorsal is up and rostral is left. Scale bar, 500 μm. White outlined boxed areas in each image are captured at high resolution to visualize regenerating CST fibers caudal to lesion for red channel only. Scale bar, 100 μm. (B) Quantification of CST labelling efficiency using anatomical tracer at the cervical region of injured spinal cord for WT (*n* = 11), E2 (*n* = 13) and E4 (*n* = 14). (C) Quantification of CST axons based on anatomical tracer in lumbar spinal cord caudal to lesion 63 days after SCI with tracer injection at day 42. CST fibers were assessed 400 μm caudal to the lesion site in WT (*n* = 39), E2 (*n* = 35), E4 (*n* = 31). (D) Quantification of spared CST axons at 10 days after SCI, traced by biotin-dextran-amine tracer injection at post-injury day 1, in lumbar spinal cord 400 μm caudal to lesion in WT (*n* = 5) and E2 (*n* = 6). (E) Quantification of serotonergic (5-HT+ve) axon length at 63 days after SCI in the ventral horn of lumbar spinal cord for WT (*n* = 14), E2 (*n* = 17), E4 (*n* =16). (F) Quantification of spared serotonergic (5-HT+ve) axon length at 10 days after SCI in the ventral horn of lumbar spinal cord for WT (*n* = 5) and E2 (*n* = 6). (G) Transverse section photomicrograph of ventral horn lumbar spinal cord at 63 days after injury stained with anti-5-HT antibody. Scale bar, 100 μm. Data shown as mean ± SEM. *p* values calculated by one-way ANOVA with Sidak’s multiple comparisons test for (B): F = 0.24, dF = 2; (C): F = 68.48, dF = 2; (E): F = 10.01, dF = 2 and by two-tailed unpaired t-test for (D): t = 0.09, df = 8; (F): t = 0.37, dF = 9.

To evaluate axonal growth as a function of ApoE, we assessed descending CST projections from motor cortex and 5-HT tracts from hindbrain raphe neurons, both of which are known to contribute to functional performance after SCI. To trace the CST unilaterally, we injected AAV-mCherry into the sensorimotor cortex 9 weeks after SCI and collected tissue 4 weeks later at 13 weeks post-SCI. There was equally robust labelling of CST axons rostral to the injury site in parasagittal sections of the thoracic spinal cord and in transverse sections of the cervical cord for the E2, E4 and WT mice (Fig. 3A and 3B). At 400 μm caudal to the injured site there were rare fibers in WT mice, numbering 0.2% of the cervical count (Axon Index of 0.002) and no fibers were detected in E4 mice (Fig. 3A and 3C). There were three times more CST fibers caudal to the injury in the E2 cohort as compared to the WT mice. Examples from multiple additional mice are shown in Fig. S3A. Caudal CST fibers in EKO and E3 mice at 13 weeks after SCI were as rare as in WT mice (Fig. S3C).

We considered whether the observed E2 CST fibers grew after injury, or were initially spared from axotomy. In separate cohorts of mice from which tissue was collected 10 days after injury, CST tracing initiated with biotin-dextran-amine injection one day after injury showed robust labelling immediately rostral to the injury, but no fibers below the injury in WT and E2 mice (Fig. 3D). Coupled with the normal development of the CST in ApoE mutant mice (Fig. S2A), these data demonstrate that the caudal CST fibers of E2 mice derive from post-SCI axon growth either via long-distance axon regeneration or via regenerative sprouting from rare uninjured fibers.

We also visualized raphespinal fibers by anti-5HT staining in the ventral horn of the lumbar enlargement. The dorsal over hemisection injury interrupts many, but not all of these axons, and in WT mice there is a reduced density of 5HT axons in the lumbar ventral gray matter (Fig. 3E and 3G). The density of raphespinal fibers is increased more than 3-fold in E2 mice as compared to WT, despite the normal raphespinal development in uninjured E2 mice (Fig. S2B). The E4 genotype had no effect on 5HT innervation density in the caudal cord after SCI. Multiple additional examples are shown in Fig. S3B. Analysis of lumbar raphespinal fibers in mice sacrificed at 10 days post-SCI showed few 5HT axons in the lumbar cord with no difference in the between genotypes (Fig. 3F). These findings are consistent with selective enhancement of distal axon sprouting in the E2 cohort at 13 weeks post-injury. We conclude that ApoE alleles influence the extent of axon regrowth and functional recovery after CNS trauma. The E2 allele promotes reparative axon growth, while E4 allele inhibits axon extension.

### ApoE isoform effects of LRP8 trafficking and signaling in axon regrowth

**Fig. 4.**
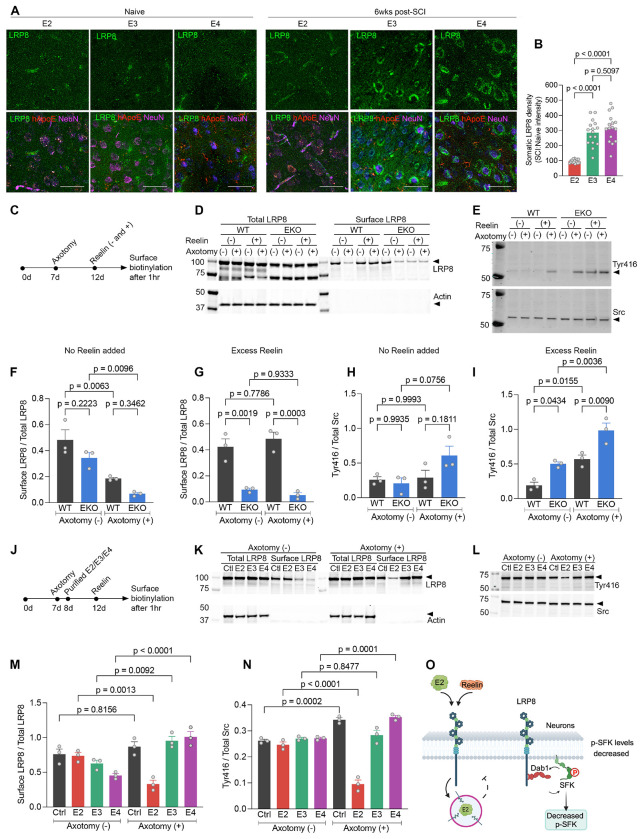
E2 impairs reelin-dependent recycling of LRP8 receptor and Src kinase activation in axotomized cortical neurons. (A) Photomicrographs of L5 from M1 brain cortex stained for anti-LRP8 (green), anti-hApoE (red) and anti-NeuN (magenta) 42 days after SCI compared to age-matched naïve controls for indicated genotypes. Scale bar, 200 μm. (B) LRP8 levels in neuronal soma at 42 days after SCI for E2, E3, E4 mice (*n* = 14). (C) Overview of study design to test influence of axotomy and reelin in LRP8 recycling and pTyr416-Src Family Kinase (SFK) activation in WT and EKO neurons. (D) LRP8 immunoblot using total and biotinylated surface protein extracts from (C) with actin as control. (E) pTyr416-SFK immunoblot using extracts from (C) with total Src as control. (F) Surface LRP8 with no added reelin from three independent experiments described in (C). (G) Surface LRP8 with excess reelin from three independent experiments described in (C). (H) Src kinase activation with no added reelin from three independent experiments described in (C). (I) Src kinase activation with excess reelin from three independent experiments described in (C). (J) Overview of study design to test influence of purified allelic ApoE lipoparticle preparations and reelin on LRP8 recycling and pTyr416-SFK activation in axotomized WT cortical neurons. (K) LRP8 immunoblot using total and biotinylated surface protein extracts from (J) with actin as control. (L) pTyr416-SFK immunoblot using extracts from (J) with total Src as control. (M) Surface LRP8 with excess reelin from three independent experiments described in (J). (N) Src activation with excess reelin from three independent experiments described in (J). (O) Schematic of cellular events in regenerating WT cortical neurons treated with E2. LRP8 undergoes rapid endocytosis in presence of reelin. Astrocytic E2 sequesters LRP8 to intracellular compartments thereby reducing downstream SFK activation in regenerating cortical neurons. Data shown as mean ± SEM and *p* values calculated by one-way ANOVA with Tukey’s multiple comparisons test for (B): F = 43.74, dF = 2; for (F): F = 16.36, dF = 3; for (G): F = 29.30, dF = 3; for (H): F = 3.47, dF = 3; for (I): F = 24.02, dF = 3; for (M): F = 15.82, dF = 7; for (N): F = 59.86, dF = 7.

Based on the cell culture axotomy data, we hypothesized that increased CST and raphespinal regrowth after injury was due to altered ApoE–LRP8 signaling in neurons subject to axotomy by SCI. We explored LRP8 subcellular localization as a marker of altered receptor signaling. The distribution of LRP8 in L5 neurons of M1 cortex from E2, E3 and E4 at 6 weeks after SCI was compared to naïve controls (Fig. 4A, 4B). While all genotypes tested had similar overall LRP8 levels in M1 cortex pre- and post-SCI, the effect of SCI was significantly different between ApoE genotypes. The E3 and E4 cortex showed a pronounced shift of LRP8 immunoreactivity from neuropil to neuronal cell soma after SCI, while an altered subcellular distribution was not detected in E2 samples. By contrast, the subcellular distribution of LRP5 and LRP6 receptors in the cerebral cortex was not altered after SCI or with different ApoE genotypes (Fig. S4A–S4D). These data demonstrate ApoE allele-specific regulation of LRP8 after SCI.

We next evaluated LRP8-related signal transduction in cultured neurons as a function of axotomy and ApoE, focusing on the status of receptor-dependent Src family kinase activation as well as cell surface LRP8 localization. We performed a cell surface biotinylation experiment in wild-type and EKO primary cortical neurons to assess surface LRP8 retention while monitoring phospho-Src family (pTyr416) levels (Fig. 4C). Cytoplasmic Src kinase activity is regulated by phosphorylation of Y527 and Y416. Y527 suppresses kinase activity whereas Y416 enhances kinase activity by stabilizing the activation loop for substrate binding. Therefore, we monitored pTyr416 levels. In WT neurons, axotomy alone led to internalization of more than 50% of LRP8 (Fig. 4D and 4F), with little change in Src activation (Fig. 4E and 4H). Deletion of ApoE synergized with the axotomy-induced LRP8 internalization, and led to increased pTyr416 levels. Since both Reelin and ApoE are ligands regulating LRP8, we included experiments with exogenous Reelin added. In WT neurons, excess Reelin prevented axotomy-induced LRP8 internalization (Fig. 4D and 4G), but supported axotomy-induced Src activation (Fig. 4E and 4I). In ApoE null cultures with Reelin added, surface levels of LRP8 were low even without axotomy, and axotomy-induced Src activation was enhanced synergistically to the highest level. Thus, there is a robust interaction of axotomy, excess reelin and ApoE deletion with regard to LRP8 internalization and Src kinase activation.

We considered how ApoE isoforms might interact with axotomy to regulate LRP8 and Src in cortical neurons. It is known that ApoE isoforms differ in their binding to LRP receptors(25, 30, 31) and in their regulation of synaptic homeostasis in AD(32–34). Therefore, we examined the effect of different purified ApoE lipoparticles on the axotomy response for both Src activation and LRP8 internalization in wild-type cortical neurons supplemented with Reelin (Fig. 4J). Without injury, excess human ApoE did not substantially alter surface LRP8 level or Src activation (Fig. 4K, 4M and 4N). With no exogenous human ApoE particles added, axotomy elevated pTyr416 phospho-Src family levels moderately, but did not alter LRP8 distribution. In the presence of added E2, axotomy reduced surface LRP8 and strongly suppressed Src activation. In stark contrast, cultures with E4 showed increased surface LRP8 and Src activation after axotomy. The effects of E3 were intermediate. In contrast to LRP8, surface localization of LRP5 and LRP6 was not regulated by axotomy of ApoE (Fig. S4E–S4H). These data demonstrate strong interactions between axotomy and ApoE isoforms to regulate LRP8 surface levels specifically and to couple with downstream Src signaling.

### Forebrain excitatory neurons show an ApoE allele-specific transcriptomic response to spinal injury

We sought to provide a comprehensive transcriptomic view of forebrain responses to SCI as a function of ApoE genotype given the evidence for greater repair and recovery in E2 mice coupled with altered LRP8 signaling. We profiled single cell transcriptomes in ApoE knock-in mice with and without SCI at a relevant time point to uncover pro-regenerative signatures. Dorsal over hemi-sections of the thoracic spinal cord of E2 and E4 mice were created, as in the behavioral and tracing studies above. Single nuclei were extracted from the forebrain region of E2 and E4 mice without SCI or at 11 dpi (days post-injury) for snRNAseq. Data from 20 mice across the four groups (E2, E4, E2-SCI, E4-SCI) passed our quality check and were used for cellular profiling. Data filtering and cell clustering retained 201,941 nuclei for assessment of cellular composition and expression (Fig. S5). To create a cellular pro-regeneration atlas, our clustering paradigm segregated 10 major cell classes consistent with standard markers: ExNeurons, InNeurons, OPCs, oligodendrocytes, astrocytes, microglia, pericytes, vLMCs, endothelial, ependymal. Within clusters, the nuclei frequencies for the four experimental groups were similar (Fig. S5). This is consistent with limited cell death or proliferation in the forebrain distant from the SCI site at this timepoint. We therefore focused on gene expression changes within cell clusters that might correlate with the differential response to injury and ApoE allele.

**Fig. 5.**
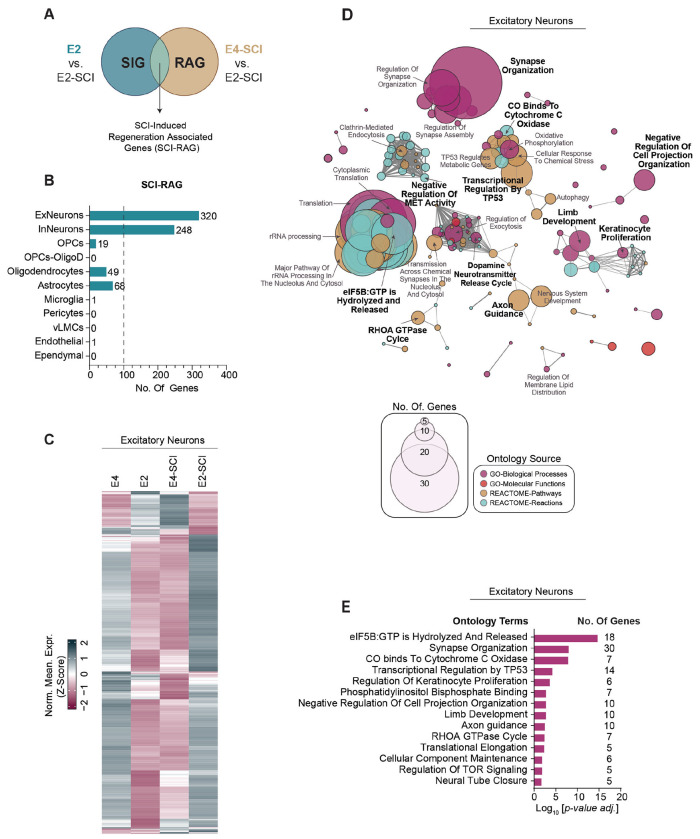
SCI-induced forebrain gene expression changes dependent on ApoE and linked to axon regeneration. (A) Schematic describing comparative analysis to identify SCI-induced regeneration-associated genes (SCI-RAGs. Genes are deemed as SCI-RAGs if they are differentially expressed post-SCI (E2 vs E2-SCI) and are associated with the post-SCI regenerative phenotypes of E2 (E4-SCI vs E2-SCI). (B) Gene count of identified SCI-RAGs across multiple cell types. The identified SCI-RAGs for pooled excitatory and inhibitory neurons listed in Supplemental Table S1. (C) Heatmap showing the relative normalized expression of SCI-RAGs of excitatory neurons. (D) Pathway enrichment analysis of excitatory neurons SCI-RAGs depicted in B and C. Enrichment terms (nodes) are organized into functional groups linked by shared gene associations (edges). Nodes are color-coded according to their respective gene ontology (GO) source and scaled in size by the number of gene-term associations. Intra-group significant terms (leading terms), in bold, are selected based on their respective *p*-values. A complete list of enriched pathway terms can be found in Supplemental Table S2. (E) Bar plot of the leading terms, their *p*-values and associated gene counts, for the pathway enrichment analysis of excitatory neuron SCI-RAGs shown in D.

To evaluate E2-specific pro-regenerative genes, we identified differentially expressed genes as SCI-induced (SIG; E2 versus E2-SCI) or regeneration-associated (RAG; E4-SI versus E2-SCI), and then focused on their intersection as SIG-RAG genes (Fig. 5). This yielded 320 SIG-RAG genes in ExNeurons and 248 in InNeurons. Strikingly, there were far fewer SIG-RAG genes in any glial cell type. While 49 and 68 genes in the SCI-RAG were identified for oligodendrocytes and astrocytes, respectively, only a single gene met these criteria in microglial cells. Thus, the primary molecular changes in the forebrain associated with greater recovery and repair in the E2-SCI mice are neuronal, and not glial. In excitatory neurons, a heatmap of SCI-RAG genes (Fig. 5C) showed that the transcriptomic response of the forebrain to SCI was largely opposite for E2 versus E4. The expression of many genes was induced in forebrain ExNeurons of the E2-SCI mice, while being suppressed in E4-SCI mice. The few genes induced in E4-SCI neurons, were suppressed in E2-SCI mice. These profiles provide a molecular correlate of the differential repair and recovery outcomes of these mice from SCI.

To characterize the cellular processes underlying reparative responses, the differentially expressed SIG-RAG genes of ExNeurons were assessed for pathway enrichment (Fig. 5D, 5E). The most prominent pathways related to neural plasticity, axon guidance and RNA metabolism. Of particular note, terms regarding synaptic organization and post-synaptic density may relate to plasticity of supraspinal systems contributing to recovery after SCI, while those related axon guidance and RHOA GTPase signaling pertain to axonal growth and neural repair. Regulation of rRNA processing may reflect a general contribution of translational regulation to the E2-SCI phenotype.

Separate analysis of the SCI category and the RAG category yielded similar conclusions to the assessment of the SCI-RAG intersection (Suppl. Fig. S6). When comparing E2-SCI to E2 mice (Fig. S6A, S6B, S6D), the most prominent changes were expression increases in ExNeurons, with few glial changes. We observed that the E2-SCI mice showed some changes in endothelial gene expression relative to uninjured E2 mice that did not meet SCI-RAG criteria. In contrast, comparison of ExNeuron expression in E4-SCI vs E4 mice, showed predominantly down-regulated genes in the mice with poor SCI recovery (Fig. S6C). Pathway enrichment of ExNeuron E2-SCI vs E2 expression again identified synapse organization, GTPase regulation and translational regulation (Fig. S6D). We also assessed differential expression between E4 and E2 mice with SCI (Fig. S6E–G). As in the other comparisons, the most striking change was downregulation of genes in ExNeuron and InNeuron of E4 (upregulation in E2), with few glial changes. The major pathways identified amongst differentially expressed genes related to synapse organization plus neuronal development and projection. Thus, regardless of pairwise comparison strategy, the E2-SCI mice with enhanced repair and recovery exhibit expression of synaptic organization and axon projection genes in neurons that E4 mice fail to upregulate after SCI.

**Fig. 6.**
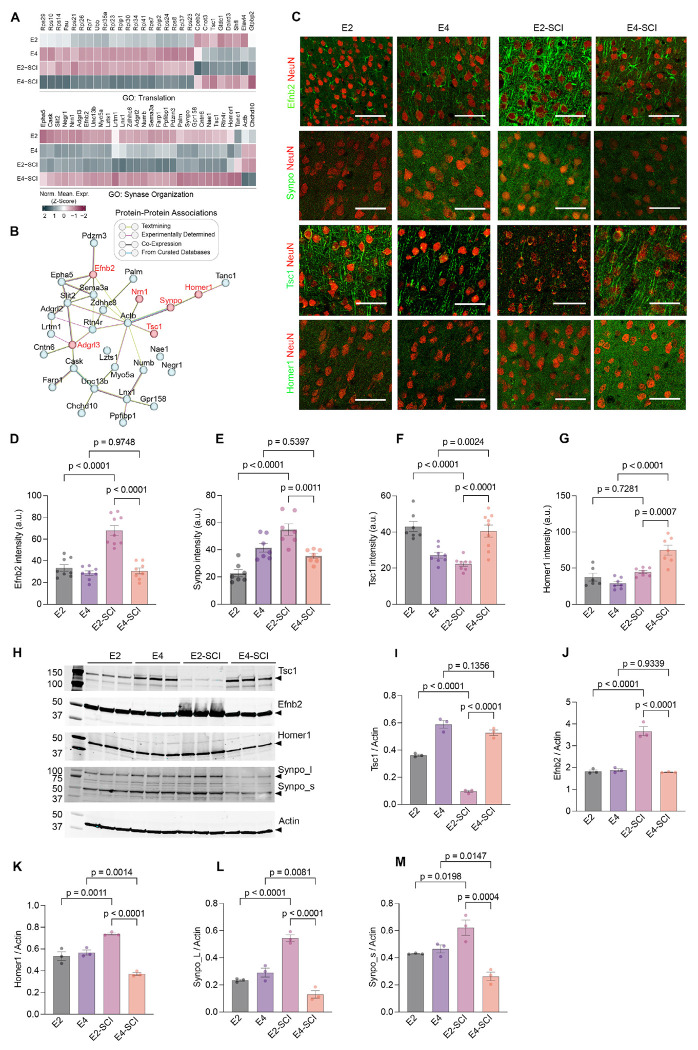
ApoE allele-specific regulation of synaptic protein expression in forebrain after SCI (A) Heatmap showing normalized gene expression changes associated with translation and synapse organization GO terms from pathway enrichment analysis performed on excitatory neurons SCI-RAGs depicted in Fig. 5D and 5E, and listed Supplemental Table S2. (B) Protein interaction network of the GO: synapse organization associated genes shown in A. Protein associations (edges) are color-coded based to the evidence source of their protein-protein association. (C) Photomicrographs of L5 from M1 brain cortex stained for Efnb2, Synpo, Tsc1, and Homer1 in green and NeuN in red, 42 days after SCI compared to age-matched naïve controls for respective genotypes. Scale bar, 100 μm. (D) Mean signal intensity of Efnb2 in L5 of M1 cortex for E2 naive (*n* = 8), E4 naive (*n* = 8), E2-SCI (*n* = 9) and E4-SCI (*n* = 9) mice. (E) Mean signal intensity of Synpo in L5 of M1 cortex for E2 naive (*n* = 7), E4 naive (*n* = 7), E2-SCI (*n* = 7) and E4-SCI (*n* = 7) mice. (F) Mean signal intensity of Tsc1 in L5 of M1 cortex for E2 naive (*n* = 7), E4 naive (*n* = 8), E2-SCI (*n* = 8) and E4-SCI (*n* = 9) mice. (G) Mean signal intensity of Homer1 in L5 of M1 cortex for E2 naive (*n* = 6), E4 naive (*n* = 7), E2-SCI (*n* = 7) and E4-SCI (*n* = 7) mice. (H) Immunoblot analysis of selected top four candidates with total protein extracted from motor cortex and subcortical regions of respective genotype with actin as loading control. (I) Densitometry analysis of Tsc1 immunoblot. (J) Densitometry analysis of Efnb2 immunoblot. (K) Densitometry analysis of Homer1 immunoblot. (L) Densitometry analysis of Synpo_L immunoblot. (M) Densitometry analysis of Synpo_s immunoblot. Data shown as mean ± SEM. *p* values calculated by one-way ANOVA with Tukey’s multiple comparisons test for (D): F = 32.61, dF = 3; (E): F = 17.94, dF = 3; (F): F = 16.54, dF = 3; (G): F = 18.46, dF = 3; (I): F = 63.45, dF = 3; (J): F = 48.61, dF = 3; (K): F = 17.31, dF = 3; (L): F = 137.8, dF = 3; (M): F = 37.71, dF = 3.

From the single cell transcriptomic profiles, we considered individual gene expression changes. The most robust individual gene changes of ExNeuron SCI-RAG genes identified amongst the pathway terms translation and synaptic organization are summarized in a heatmap (Fig. 6A). Notably, many of the synapse organization genes have evidence for protein-protein interactions with one another (Fig. 6B). We sought to validate a subset of these changes at the protein level in cerebral cortex by immunohistology and immunoblot using samples collected 6 weeks after SCI. The protein subset to be tested was based on possession of a known function and antibody availability. Immunohistological analysis of expression in NeuN-positive neurons of L5 in M1 cortex for Efnb2 and Synaptopodin matched the forebrain ExNeuron mRNA expression pattern with highest levels in E2-SCI mice (Fig. 6C–6E). This supports their potential role in modulating repair and plasticity in CST neurons of E2-SCI mice. The patterns for Tsc1 (Fig. 6C and 6F) and for Homer1 (Fig. 6C and 6G) showed statistically significant differences between groups with lower levels in E2-SCI than E4-SCI L5-M1 neurons. The differences between immunohistology and transcriptomics for Tsc1 and Homer1 may reflect changes unique to timing (6 weeks vs 11 days) or to region (L5-M1 vs anterior forebrain). Of note, the suppression of Tsc1 has been linked to the PTEN pathway and to increased axon regeneration in simple model systems(13, 35). Immunoblot of cortical protein levels were also completed for Tsc1, Efnb2, Homer1 and Synaptopodin (Fig. 6H–6M). The biochemical studies confirmed the regulation of these proteins by ApoE allele and SCI and confirmed the histological findings in all cases, with the exception of Homer1 in E4-SCI samples. Overall, the protein analysis verified the ApoE2- and SCI-dependent expression of axon and synapse regulating pathways in the cortical neurons relevant to SCI repair and recovery.

### Therapeutic expression of E2 promotes neural repair in mice and human neurons

**Fig. 7.**
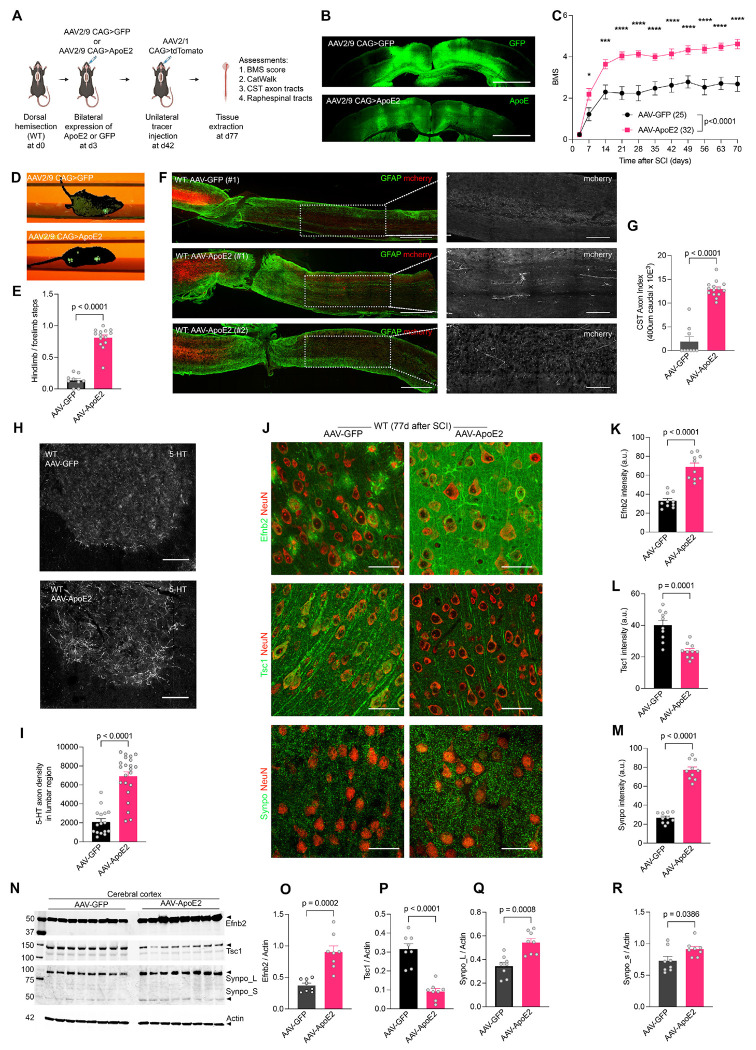
AAV-mediated expression of ApoE2 promotes functional and anatomical recovery of WT mice after SCI. (A) Overview of experimental approach to evaluate therapeutic benefit of ApoE2 in WT mice with dorsal thoracic over-hemisection injury. (B) Photomicrograph of brain cortex region of mice that received bilateral AAV2/9 injection into M1 cortex. Sections stained for anti-GFP and anti-hApoE at d77 post-injection. Scale bar, 500 μm. (C) BMS open-field locomotion assessment of WT-SCI mice receiving AAV-GFP (*n* = 25) and AAV-ApoE2 (*n* = 32). Performance scores were recorded for each animal every consecutive week. Data shown as mean ± SEM and analyzed using repeated measure multi-point ANOVA across time series followed by post-hoc Tukey’s multiple comparisons test between genotypes at indicated time points. ****p<0.0001, ***p<0.001, **p<0.01 significant difference between genotypes. (D) Single frame CatWalk video showing fore and hindlimb paw footprints in green for WT-SCI mice injected with AAV-GFP or AAV-ApoE2, and observed at d77 after SCI. (E) Limb coordination index for WT-SCI mice treated with AAV-GFP (*n* = 9) or AAV-GFP (*n* = 14), d77 after SCI. (F) Sagittal low-power photomicrographs of spinal cord around the lesion site in AAV-injected WT mice at d77 after SCI. Sections were stained with anti-GFAP (green) and anti-mCherry (red). Dorsal is up and rostral is left. Scale bar, 500 μm. White outlined boxed areas in each image are captured at high-resolution to visualize regenerating CST fibers caudal to lesion for red channel only. Scale bar, 100 μm. (G) Quantification of CST axon index in the lumbar spinal cord caudal to lesion. CST fiber numbers were measured at 400 μm caudal to the lesion site in WT mice expressing GFP (*n* = 9) or ApoE2 (*n* = 14) at 77 days after SCI. (H) Transverse axis photomicrograph of ventral horn lumbar spinal cord caudal to lesion stained with anti 5-HT. Scale bar, 100 μm. (I) Quantification of 5-HT+ve serotonergic fiber density in ventral horn of lumbar spinal cord for AAV-GFP (*n* = 16) and AAV-E2 (*n* = 22) mice at d77 after SCI. (J) Photomicrographs of L5 from M1 brain cortex stained for Efnb2, Synpo, Tsc1 in green and NeuN in red, for WT-SCI mice treated with AAV-GFP (*n* = 10) and AAV-ApoE2 (*n* = 10). Scale bar, 100 μm. (K) Mean signal intensity of Efnb2 in L5 of M1 cortex for therapeutic WT-SCI mice. (L) Mean signal intensity of Tsc1 in L5 of M1 cortex for therapeutic WT-SCI mice. (M) Mean signal intensity of Synpo in L5 of M1 cortex for therapeutic WT-SCI mice. (N) Immunoblot of selected proteins in extracts from cerebral cortex of respective genotypes with actin as loading control. (O) Densitometry analysis of Efnb2 immunoblot. (P) Densitometry analysis of Tsc1 immunoblot. (Q) Densitometry analysis of Synpo_L immunoblot. (R) Densitometry analysis of Synpo_s immunoblot. Data shown as mean ± SEM. *p* values calculated by two-tailed unpaired t-test for (E): t = 10.9, df = 21; (G): t = 9.7, dF = 11; (I): t=3.22, df=13; (K): t = 7.3, df = 18; (L): t = 4.8, df = 18; (M): t = 13.6, df = 18; (O): t = 5.4, df = 14; (P): t = 6.9, df = 14; (Q): t = 8.8, df = 14; (R): t = 4.2, df = 14.

Given the prominent effect of ApoE alleles on repair, recovery and gene expression after SCI, we hypothesized that ApoE2 expression after SCI would confer a therapeutic benefit. We introduced AAV-ApoE2 versus AAV-GFP into wild type mice at 3 days after SCI using bilateral intraparenchymal injection into cerebral cortex to evaluate this intervention (Fig. 7A). Prior to SCI efficacy studies, we evaluated the level and distribution of ApoE2 expression by immunoblot and immunohistology in uninjured wild-type, EKO and E2 mice at 28 days post-injection. As detected by a human-specific ApoE antibody, the forebrain level of ApoE2 in AAV-ApoE2 injected wild-type, EKO was equivalent to the uninjected ApoE2 knock-in mice (Fig. S7A). For ApoE2 mice, the AAV injection increased forebrain protein level by about two-fold. We also assessed the distribution of human ApoE2 protein throughout the neuroaxis histologically. No staining signal was observed in uninjected wild-type or EKO samples, validating the specificity of the method. After cortical AAV-ApoE2 injection, there was prominent human ApoE in the cerebral cortex, similar to GFP expression from AAV-GFP (Fig. 7B). In addition, the secreted human ApoE2 protein diffused widely in the CNS with strong staining observed in spinal cord neurons where cortical AAV-GFP produced no GFP signal (Fig. S7B). Thus, cortical AAV-ApoE2 provided effective delivery of the protein broadly within the mouse CNS.

The functional role of human ApoE2 overexpression was assessed in adult wild-type mice with thoracic dorsal over-hemisection injury, the same traumatic lesion utilized for the ApoE knock-in and knockout studies described above. On the third day after SCI, all mice were incapable of locomotor function with BMS scores of 1 or less. The animals were randomized to receive AAV-ApoE2 or control AAV-GFP virus at that point. The degree of locomotor recovery as measured by BMS in the open field was significantly greater in the AAV-ApoE2 group (at 70 days, 4.70 ± 0.24, mean ± SEM, n=32) than in the AAV-GFP group (2.44 ± 0.36, n=25) (Fig. 7C). Nearly all AAV-ApoE2 showed occasional or consistent plantar stepping with weight support, while most control AAV-GFP mice exhibited ankle movement but not plantar stepping or weight support. In CatWalk analysis of walking along a linear track, both limb coordination and hindpaw footprints were consistently detected in ApoE2-treated compared to GFP group (Fig. 7D). Automated analyses detected hindlimb steps for over 75% of forelimb cycles in the AAV-ApoE2 group, while recognizing hindlimb steps associated with fewer than 10% of forelimb steps by the AAV-GFP mice (Fig. 7E). After 8 weeks survival, spinal cords were collected for histology. The E2-treated group showed a greater than four-fold increase in caudal CST fibers (Fig. 7F–7G) and two-fold increase of raphespinal axon length in the ventral horn of lumbar spinal cord compared to GFP-treated group (Fig. 7H and 7I). Multiple additional examples are shown in Fig. S7C–S7D).

We sought to confirm whether AAV-ApoE2 expression after SCI generated the same molecular profile as observed for constitutive ApoE2 knock-in SCI mice. The differentially expressed proteins from SIG-RAG analysis of knock-in mice were assessed in the therapeutic AAV cohort by immunohistology and immunoblot. Localization (Fig. 7J–7M) and cortical expression (Fig. 7N–7R) changes of Efnb2, Tsc1 and Synpo in NeuN-positive L5 neurons of M1 cortex matched with the ApoE2 knock-in expression changes. These results highlight the importance of axon and synaptic plasticity pathways for therapeutic benefit of ApoE2 after SCI. Overall these results demonstrate post-injury delivery of an ApoE2 based therapeutic improves SCI recovery and neural repair.

We considered whether human neurons as well as mouse neurons were responsive to the pro-regenerative benefit of ApoE2. We examined axon regeneration from iPSC-derived glutamatergic neurons derived from stable integration of neurogenin-2 under doxycycline-inducible promoter (i^3^Neurons)(36). Twenty-four hours after axotomy of mature neurons on the 50^th^ day post differentiation, AAV-E2 or AAV-GFP was added to these *APOE3* homozygous neuronal cultures (Fig. S7G). A substantial increase of both cellular and secreted ApoE protein was confirmed for the neurons treated with AAV-E2 (Fig. S7E and S7F). Axon regeneration over the subsequent 7 days was twice as extensive for the cultures expressing ApoE2 as compared to those expressing GFP (Fig. S7H–S7I). These data support the potential therapeutic benefit for ApoE2 overexpression after CNS trauma.

## DISCUSSION

The central finding of this study is that neurons exposed to secreted ApoE2 exhibit regenerative axon growth after axotomy, a process dependent on LRP8-mediated signaling. In contrast, exposure to ApoE4, or lack of ApoE, limits axon regeneration. After spinal cord injury, the presence of ApoE2 before or after trauma supports greater corticospinal and raphespinal axon growth, improved behavioral recovery, and altered neuronal transcriptomes. Axotomy and reelin trigger LRP8 redistribution and downstream kinase activation, while ApoE2 antagonizes these effects and supports injury-induced upregulation of synapse organization genes.

A broad range of pathways have been implicated in axon regeneration using various model systems(37–42). ApoE transcription is known to modulate dorsal root ganglion sensory axon outgrowth(43). In contrast, genetic linkage studies in humans tracking recovery after traumatic CNS injury have thus far identified only a few genetic variations influencing repair or recovery(19, 20, 44, 45). Here, we investigated the ApoE–LRP8 axis, since the LRP8 receptor was a candidate from our *in vitro* axon regeneration screening(10), and genetic variation in its ApoE ligand has been linked to the outcome of human TBI recovery(19). Convergent discovery of this ligand receptor pair across the translational spectrum emphasizes the potential importance of these molecules.

The ApoE action studied here appears to be selective for neurons. The culture system includes predominantly neurons and secreted ApoE from astrocyte cultures transferred activity to ApoE knockout neuronal cultures. *In vivo* spinal cord injury studies showed robust effects on regenerative axon growth and pronounced interactions of spinal cord injury and ApoE alleles on neurons distant from the trauma site. It is abundantly clear from studies of neurodegeneration that ApoE alleles differentially affect glial cells, with alterations in microglial and astroglial metabolism, reactivity and function(29, 32, 33, 46–49). In addition, ApoE regulates macromolecular transport and metabolism to participate in neurodegenerative mechanisms(50). In the studies here, while the source of ApoE is likely to be predominantly glial, there is no evidence that glial reactions at the injury site or in the forebrain provide a substantial contribution to the improved axon growth and functional recovery. In particular, tissue sparing and glial reaction were not altered by different ApoE alleles. Many fewer differentially expressed genes were observed for microglia and astrocytes than for neurons in the brain after injury. Although ApoE’s most studied effect is on Aß accumulation and Alzheimer’s risk, our SCI and axon regeneration models show no evidence for Aß accumulation or altered APP.

The analysis of LRP8 subcellular localization and signal transduction delineate ApoE allele specific actions. Reelin and axotomy share actions to decrease surface LRP8 and increase Src family kinase activation. ApoE2, but not ApoE4 or ApoE3, antagonizes the action of axotomy and excess reelin. These data are consistent with previous descriptions of antagonistic action of ApoE and reelin for Lrp8 without axotomy(51). SCI in the presence ApoE2 leads to increased expression of multiple genes and proteins associated with synaptic organization. Gene products such as Efnb2, Synaptopodin, and Homer1 are likely to contribute to neural repair. Their increased expression is consistent with the formation of new and plastic synaptic connections. The suppression of Tsc1 in ApoE2 – SCI mice is consistent with an increased intrinsic axon regenerative growth state(13, 35). Overall, our SCI and transcriptomic studies highlights a broad cellular heterogeneity of forebrain neurons in synaptic modulation of spinal circuits for recovery in response to secreted ApoE along the neuroaxis. The relative importance of different circuits is not studied here, but likely many different neuronal populations participate in addition to corticospinal and raphespinal neurons.

One implication of these studies is that ApoE genotype can alter the prognosis for recovery from CNS trauma, likely both SCI and TBI. Worse outcomes for ApoE4 cases of SCI have been reported(20–23), though a potentially beneficial effect for the rarer ApoE2 allele has not been described. Consideration of ApoE genotyping to increase prognostic accuracy and to stratify clinical trials of various interventions may be warranted(52).

We utilized AAV injection to overexpress ApoE2 in wild type mice beginning three days after injury in a therapeutically relevant design and observed improved function and axon growth. Future experiments with contusive injury, longer delays to treatment, and systemic vectors will explore the interventional potential of this approach. In addition, kinases downstream of Lrp8 may be critical for ApoE2 benefit such that small molecule inhibitors may provide alternate sites for intervention.

These findings also have important implications for the role of ApoE in neurodegeneration. ApoE expression, and ApoE2 in particular, may be expected to promote compensatory mechanisms, through neuronal plasticity and axonal sprouting, to mitigate symptoms in Alzheimer’s disease and related disorders. Specifically, ApoE genotype may modulate progression rate in Alzheimer’s disease via compensatory mechanisms unrelated to the risk of disease or the accumulation of Aß and tau or inflammation. Thus, the role of ApoE genotype in neuronal resilience may be a critical, but still understudied, aspect of neurodegeneration. In this regard, our data from axotomy studies indicate that ApoE2 will be most beneficial while ApoE4 is most detrimental. The spinal trauma model indicates that suppression of all ApoE expression is deleterious for compensatory repair after CNS damage.

It is known that many cases of dementia are multifactorial, with vascular disease frequently co-occurring with Alzheimer’s pathology. Disruption of neuronal connectivity due to vascular compromise of white matter may respond to ApoE genotype in the same manner that disconnection due to spinal cord trauma does. Regardless of whether neuronal connectivity is damaged by vascular insufficiency, neurodegenerative protein accumulation or trauma, we expect ApoE signaling to be a critical determinant of the success of neural repair and symptomatology.

In conclusion, ApoE2 has a robust effect to increase adult CNS axon growth after trauma that is dependent on altered LRP8 signaling with increased expression of synapse organizing genes. ApoE action is predominantly neuronal in this condition. Allele-specific ApoE expression alters functional recovery after traumatic SCI in mice, with overexpression of ApoE2 improving locomotor recovery.

## Supplementary Material

1
