# Supplementary material for: Axon Regeneration and Functional Recovery after Spinal Cord Injury is Enhanced by Allele-Specific ApoE Neuronal Action through LRP8
Source: bioRxiv. 2025 Oct 13:2025.10.10.681747. Preprint. [Version 1] doi: 10.1101/2025.10.10.681747 (PMC12632883; doi:10.1101/2025.10.10.681747)
Supplement: 1 [file NIHPP2025.10.10.681747v1-supplement-1.pdf]

bioRxiv preprint doi: https://doi.org/10.1101/2025.10.10.681747; this version posted October 13, 2025. The copyright holder for this preprint (which was not certified by peer review) is the author/funder, who has granted bioRxiv a license to display the preprint in perpetuity. It is made available under aCC-BY 4.0 International license.

SUPPEMENTARY MATERIALS

The PDF file includes:

Materials
Figs. S1 to S7

METHODS

Animals

Spinal cord injury study was performed in mice with following genotypes - C57BL/6J (JAX stock#000664), ApoE KO (JAX stock #002052), ApoE2 KI (JAX stock #029017), ApoE3 KI (JAX stock #029018), ApoE4 KI (JAX stock #027894) and Lrp8 KO(*53*) (JAX stock #003524). Mice were maintained on a 12-h light/dark cycle with regular food and water under 40%-60% humidity. All animal procedures were performed following institutional guidelines and regulations. To minimize investigator bias, the study was blinded during entire analysis.

Primary neuronal cultures

Primary mouse cortical neurons were cultured in Neurobasal supplemented with B27 (10X) and L-glutamine (2 mM) while iPSCs were maintained in Essential 8 Medium (Gibco A1517001) and iPSC-derived glutaminergic human neurons were maintained in DMEM/F-12 based medium supplemented with essential growth factors described in detail below. All cells were maintained at 37°C with 5% CO_2.

Cell culture and transfection

HEK293T (ATCC, CRL11268) cells were cultured in DMEM/F-12 (Gibco 11320-033). To ensure absence of mycoplasma, all cell lines were periodically examined using LookOut Mycoplasma PCR kit (Sigma-Aldrich, MP0035). A day before transfection, HEK293T cells were passaged at a density of 10^7 cells/15-cm plate and transfected at 80% confluency with respective constructs for AAV production. DNA: PEI transfection mixture containing 150 µl of polyethylenimine (PEI, Polysciences Inc.) incubated at RT for 15 min before adding to HEK293T cells. All cells were maintained at 37°C with 5% CO_2.

Mouse behavioral tests

Two researchers unaware of the mice genotype performed all behavioral tests. We used Basso Mouse Scale (BMS) as a measure of open-field locomotion(*54*). BMS has a quantitative scale from 0 to 9. BMS scoring were done once pre-injury and starting on 3d of injury and weekly thereafter for all dorsal thoracic over-hemisection experiments.

The CatWalk XT (v 10.6) gait analysis system (Noldus, Netherlands) design consisted of a 1.5 m black corridor walkway on a black glass plate using green LED bottom lighting. Entire system is placed in dark silent room. Paw prints were captured by 100 fps high-speed camera positioned beneath the black glass floor. Walkway area and compliance parameters are set following company recommendations. Mice were habituated to the system and the walkway voluntarily, twice a day for three days to complete three accomplished runs. At the end of training period, mice were tested by crossing the corridor three times. To detect the paws from the background, we used same detection settings for all genotypes (camera gain: 20.50, green walkway light (15.5, green intensity threshold: 0.15, red ceiling light :17.3). To measure the limb coordination, we measured fore-hind step ratio using number of fore and hind limb steps taken by each mouse across three replicate runs.

Surgical procedures for SCI, CST tracing and CNS gene therapy experiments

All animal procedures and post-operative care were performed in accordance with the Institutional Animal Use and Care Committee guidelines at Yale University. Age-matched adult (12-13 weeks) female mice were subjected to dorsal thoracic over-hemisection (75% depth) as described previously(*55*). All animals received subcutaneous injection of 100 mg/kg ampicillin and 0.1 mg/kg Buprenex twice a day for the first 2 d after surgery and additional injections later as necessary.

To trace CST tracts, we unilaterally injected AAV9-CAG>tdTomato (Addgene #59462) into sensorimotor cortex to anterogradely label the CST at each of the five sites (coordinates from bregma in mediolateral/anterior–

bioRxiv preprint doi: https://doi.org/10.1101/2025.10.10.681747; this version posted October 13, 2025. The copyright holder for this preprint (which was not certified by peer review) is the author/funder, who has granted bioRxiv a license to display the preprint in perpetuity. It is made available under aCC-BY 4.0 International license.

posterior format in mm: 1.0/0.0, 1.5/1.5, 1.5/0.5, 1.5/−0.5, 1.5/−1.5) for a total of 1.5 µl volume. Mice were kept for additional 4 weeks before being euthanized for morphometric analysis. For expression of ApoE2 gene therapy experiments, AAV9-CAG-ApoE2 and control AAV9-CAG-GFP (Addgene #37825) was injected bilaterally into sensorimotor cortex (coordinates as mentioned above) at 3 d after dorsal hemisection procedure. All animals underwent surgery received subcutaneous injection of 100 mg/kg ampicillin and 0.1 mg/kg Buprenex twice a day for the first 2 d after surgery and additional injections later as necessary. The study was randomized and blinded for genotypes and virus used.

AAV-ApoE2 vector construction and AAV9-ApoE2 virus production

The coding sequence for human ApoE2 was amplified from pCMV4-ApoE2 (Addgene#87085) and inserted at BamH1 and HindIII sites of pAAV-CAG (Addgene#59462) upstream of WPRE and SV40 pA sequence by replacing tdTomato coding region in #59462. ApoE2 fragment was amplified using Q5^{R} Hot Start High-Fidelity 2X master mix (# M0491L) according to manufacturer protocol with ApoE-specific primers: forward primer (20mer): ATGAAGGTTCTGTGGGCTGC and reverse primer (20mer): GCAGCCCACAGAACCTTCAT. The final vector pAAV-CAG>hApoE2-WPRE-SV40pA was sequence verified in-house using internal and vector specific sequencing primers.

A day before transfection, HEK293T cells were passaged at a density of 10^7 cells/15-cm plate and transfected at 80% confluency. For AAV production, the plasmid cocktail containing - pDF6 helper (18 µg of Addgene#112867), pAAV-CAG>ApoE2 (6 µg), AAV packaging plasmid expressing Rep/Cap genes pAAV2/9n (6 µg of Addgene#112865) were prepared in 3 ml of serum-free DMEM. DNA:PEI transfection mixture containing 150 µl of polyethylenimine (PEI, Polysciences Inc.) was incubated at RT for 15 min before adding to HEK293T cells. After 96 h following transfection, the cells were harvested and treated with DNase1 (10 U/ml, AMPD1, Sigma-Aldrich) and benzonase (50 U/ml, #70746 Millipore) for 40 min at 37°C. The mixture was centrifuged at 3,000 x *g* for 20 min at 4°C to remove cell debris. The supernatant containing viral particles were further purified by ultra-centrifugation using iodixanol density gradient(*56*). To estimate the viral titers samples were compared against a standard curve generated from known titer diluted from 10^8 to 10^{13} genome copies per ml. Viral titers were determined using iQ SYBR Green Supermix (Bio-Rad) and quantitative PCR (Bio-Rad CFX96). Average AAV9-ApoE2 viral titers used for *in vitro* transduction of iPSC derived human neurons were 10^{10}-10^{11} genome copies per ml and 10^{11}-10^{12} genome copies per ml for *in vivo* CNS transduction experiments.

Primary mouse cortical neuron culture

We used postnatal d1 pups (P1) from respective genotypes to dissect cortices in ice-cold BrainBits medium. Enzymatic dissociation in 1X HBSS (Mg/Ca-free) supplemented with 100 U/ml DNAse (04716728001, Roche) containing Papain (20 U/ml, Worthington LK003178), 0.5M EDTA and 1 mM $CaCl_2$ at 37°C for 30 min followed by mechanical dissociation. Cells were counted using hemocytometer and diluted to achieve a seeding density of 2.5-4.0 x 10^4 per 200 µl for each well and plated on Corning BioCoat poly-D-Lysine coated glass 96-well plates (#354461) for axon regeneration assays and 12-well plates (#354470) for biochemistry experiments. Cultures were kept in 37°C with 5% CO2 incubator in Neurobasal-A media supplemented with 0.5% penicillin/streptomycin, 0.5% B27 and 2 mM Glutamax.

iPSC maintenance and differentiation of glutamatergic human neurons

Engineered iPSC expressing mammalian NGN2 (neurogenin 2) under doxycycline-induced system in the AAVS1 safe harbor locus, termed as GMK2 iPSC cell line also express inducible CRISPR interference machineries (pC13N-dCas9-BFP-KRAB). The parental human iPSC cell line used was CRISPRi-i3N iPSCs(*36*). The aliquots of the frozen cells in clumps were thawed at 37°C water bath and resuspended in Essential 8 Medium (GIBCO/Thermo Fisher Scientific; Cat. No. A1517001) containing 10 nM ROCK inhibitor Thiazovivin (#72254, Stem Cell Technologies) [E8+T]. iPSCs were centrifuged 350 x *g* at 4°C. The pelleted cells were resuspended in [E8+T] and platted in 1-well of a Vitronectin (#A31804, Vitronectin (VTN-N) Recombinant Human Protein, Truncated, GIBCO /ThermoFisher) coated 6-well plate. Cells were allowed to grow for 3-4 d or at 70-80% confluency to form the iPSC clumps with everyday [E8+T] media exchange. 70-80% iPSC cell culture was then split with Gentle Dissociation reagent (GDR) and again plated as clumps containing E8 medium in the vitronectin coated 6 well plates. In this way the iPSCs were maintained in a pluripotent state.

bioRxiv preprint doi: https://doi.org/10.1101/2025.10.10.681747; this version posted October 13, 2025. The copyright holder for this preprint (which was not certified by peer review) is the author/funder, who has granted bioRxiv a license to display the preprint in perpetuity. It is made available under aCC-BY 4.0 International license.

iPSCs were then allowed to grow (70-90%) confluency to differentiate and induce iPSCs into glutamatergic neurons. iPSCs were released by incubating the cells at *37*°C for 7 mins with StemPro Accutase Cell dissociation reagent (GIBCO Cat No: A11105-01) and centrifuged at 250 x *g* at 4°C for 5 mins. Next, the pelleted iPSC cells were resuspended in N2 pre-differentiation medium containing knockout DMEM/F12 (GIBCO/Thermo Fisher Scientific; Cat. No. 12660-012), 1X MEM non-essential amino acids (GIBCO/Thermo Fisher Scientific; Cat. No. 11140-050), 1X N2 supplement (GIBCO/Thermo Fisher Scientific; Cat. No. 17502-048), 10 ng/mL NT-3 (PeproTech; Cat. No. 450-03), 10 ng/mL BDNF (PeproTech; Cat. No. 450-02), 1 µg/mL mouse laminin (Thermo Fisher Scientific; Cat. No. 23017-015) and 10 nM ROCK inhibitor Thiazovivin (#72254, Stem Cell Technologies) to induce mNGN2 expression. Pelleted iPSCs were counted and plated at 7 x 10^5 cells per well in a matrigel-coated 6-well plate contained 2 ml of N2 pre-differentiation medium per well. iPSC cells were grown in single cells for three days in N2 pre-differentiation medium. Three days later, as d0, the pre-differentiated cells were released by incubating the cells at 37°C for 7 mins with StemPro Accutase Cell Dissociation Reagent (GIBCO Cat No: A11105-01) and then centrifuged at 250 x *g* at 4°C for 5 mins. The pelleted pre-differentiated cells were then resuspended in classic neuronal medium containing the following: half DMEM/F12 (GIBCO/Thermo Fisher Scientific; Cat. No. 11320-033) and half Neurobasal-A (GIBCO/Thermo Fisher Scientific; Cat. No. 10888-022) as the base, 1X MEM Non-Essential Amino Acids, 0.5X GlutaMAX Supplement (GIBCO/Thermo Fisher Scientific; Cat. No. 35050-061), 0.5X N2 Supplement, 0.5X B27 Supplement (GIBCO/Thermo Fisher Scientific; Cat. No. 17504-044), 10 ng/mL NT-3, 10 ng/mL BDNF, 1 µg/mL Mouse Laminin, and 2 µg/mL doxycycline hydrochloride. Pre-differentiated cells were counted and plated at (5,000-10,000) cells per well of a BioCoat Poly-D-Lysine 96-well plate (Corning; Cat. No. 356640) in 100 µl of Classic Neuronal Medium per well. After 7 DIV, half of the classic neuronal medium was removed, and an equal volume of fresh medium was added without doxycycline. At 14 DIV, half of the medium was removed and twice that volume of fresh medium without doxycycline was added. At 21 DIV, one-third of the medium was removed and twice that volume of fresh medium without doxycycline was added. At 28 DIV and every week after, one-third of the medium was removed and an equal volume of fresh medium without doxycycline was added. In this way the neurons were allowed to grow for 45-50 days.

Astrocyte conditioned medium (ACM)

Wild-type, EKO and E2/E2/E4 KI mouse cerebral cortices were removed from postnatal d1 pups (P1) in ice-cold BrainBits medium. McCarthy and deVellis (MD) astrocytes protocol was adopted for culturing MD astrocytes(*57*). Enzymatic digestion performed with 2 ml of 0.25% trypsin at 37°C for 20 min followed by 1 ml of DMEM+FBS to deactivate trypsin. After adding 0.05 mg/ml DNase, the tube was spun at 300 x *g* for 5 min and the cell pellet was re-suspended in 1 ml of fresh DMEM+FBS. This suspension was then filtered through 70 µm cell strainer and the cells were plated onto an uncoated plastic T-25 flask maintained in a rotary shaker at 200 rpm. After 3 DIV, old medium was replaced with astrocytes conditioned medium (ACM) containing 50% neurobasal + 50% DMEM without phenol red, glutamine, pyruvate, N-acetylcysteine (NAC) and penicillin-streptomycin. Cultures were then maintained for additional 5 d to enrich secreted factors from astrocytes. Thereafter, any dead cells and debris were removed by centrifugation at 3,000 x *g* for 30 min. ACM was concentrated with a 3kDa MWCO (Amicon, 15ml) at 3,000 x *g* for 15 min at 4°C. After Bradford estimation of total protein concentration in ACM, aliquots were stored at -80°C for further experiments.

Isolation of native ApoE lipoprotein particles from ACM

We coupled a mouse monoclonal WUE-4 apoE antibody that detects the three isoforms of human ApoE (NB110-60531) or mouse IgG isotype control to CNBr-activated Sepharose 4 fast flow beads for isolation of native ApoE lipoprotein particles. Antibody-coupled beads were washed with coupling buffer (0.1 M $NaHCO_3$, 0.5 M NaCl, pH 8.3) and the unreacted groups were quenched with 0.1 M Tris-HCl, pH 8.0 with rocking for 2h at 20°C. Beads were further washed with 0.1 M Tris-HCl pH 8.0 with 0.5 M NaCl and finally the antibody-conjugated beads were washed with 1X PBS prior to immunoprecipitation of ApoE lipoprotein particles from ACM. Concentrated ACM was then incubated overnight at 4°C with antibody-conjugated beads with end-to-end mixing and mild agitation. The beads were washed in 0.5 M NaCl to remove nonspecific proteins and the native apoE lipoprotein particles were eluted using 3 M NaSCN for immunoblot analysis of native PAGE gel with human anti-ApoE (1:500 #ab52607).

Brain and spinal cord tissue processing

bioRxiv preprint doi: https://doi.org/10.1101/2025.10.10.681747; this version posted October 13, 2025. The copyright holder for this preprint (which was not certified by peer review) is the author/funder, who has granted bioRxiv a license to display the preprint in perpetuity. It is made available under aCC-BY 4.0 International license.

Four weeks after AAV injections, mice were euthanized with isoflurance and transcardially perfused with PBS followed by 4% PFA in PBS. Brains and spinal cords were dissected, embedded in 10% gelatin and postfixed in 4% PFA overnight at 4°C. Next day, gelatin embedded tissues were transferred to PBS with 0.05% sodium azide and stored at 4°C. Brains were then processed using vibratome (Leica VT1000S). Coronal sections (40 µm) of brain were processed using vibratome (Leica VT1000S) and stored in PBS with 0.05% sodium azide at 4°C. For spinal cord, we generated transverse or sagittal sections depending on the analysis. We used sagittal sections (40 µm) for scoring 5-HT raphespinal in lumbar caudal to injury site and CST tract labeling density at cervical region. Furthermore, a 10 mm block of spinal cord consisting of thoracic hemisection site (-5 mm rostral and +5 mm caudal) was excised and sectioned sagittal (40 µm) to access CST tract regeneration and severity of lesion. To analyze CST regeneration, sections underwent an immunohistochemistry protocol to enhance tdTomato signals for confocal microscopy. In brief, sections were incubated for 10 min in 0.3% H_2O_2 at room temperature, washed thrice in PBST (0.05% Triton-X) and blocked in 2% pre-filtered BSA with 5% normal donkey serum (Jackson ImmunoResearch: 017-000-121) and 5% normal goat serum (005-000-121) for 1 h at room temperature. Sections were then incubated overnight in Rb anti-mCherry (1:1000; ab167453) prepared in blocking solution mentioned above. Following day, the sections were washed 3 times in PBS-T and then incubated for 2 h in 568 D-anti-Rb (1:1000). For scoring raphespinal axons, sagittal sections were blocked as mentioned above and stained with Rb anti-5-HT serotonin 1A receptor (1:10000; Immunostar 24504). For scoring glial scar at injury site, the following antibodies were used monoclonal anti-GFAP 2.2B10 (1:1000 #13-0300); polyclonal anti-Iba1 (1:500 Wako); polyclonal anti-Laminin (1:1000 #PA1-167730). For brain coronal sections to analyze CST cell bodies in M1 cortex, following antibodies were used anti-Lrp8 (1:500 #4H3E6 MABN1872); human anti-ApoE (1:500 #ab52607); anti-NeuN (1:1000 A60 #ABN91); anti-ChAT (1:1000 AB143); anti-LRP5 (1:1000 36-5400); anti-LRP6 (1:2000 # PA5-101047); anti-Efnb2 (1:1000 # MA5-32740); anti-Tsc1 (1:1000 # 6935); anti-Synpo (1:500 # 163 002); anti-Homer (1:1000 # 160 023). After 3x washes in PBS-T, every other section was mounted on Superfrost Plus slide with VectorShield antifade medium (H-1200-10). At the end, fluorescent Nissl stain performed for one h at room temperature using NeuroTrace 435/455 Blue (1:1000, N21479). Sections were imaged birectional at 40x magnification using Leica SP8 Confocal at 1024-pixel resolution with 400 scan speed. Images were acquired titled and stitched using Leica SP8 post processing software for further morphometric analysis. The number of regenerating fibers in spinal cord sections were normalized to dorsal column CST bundle at level of cervical enlargement. Axonal counting and glial scar quantification was performed as previously described(*55*). Animals are chip-tagged and respective IDs were used for sampling and data collection and hence performed blind to genotypes.

Intensity measurements

For quantification of somatic Lrp8 signal intensity, we used ImageJ/FIJI(*58*). For each condition, we used *z*-projection of deconvolved stacks for two NeuN and Lrp8 channels. Using grey scale images, a nuclear mask was generated using NeuN pattern. The nuclear mask was then extracted and transferred to grey scale images of Lrp8 images and dilated twice to cover the Lrp8 signal distribution in each neuron. The difference between nuclear mask and twice dilated mask represents Lrp8 intensity in each soma. Instead of using cytoplasmic area by selecting the whole neuronal soma, here we use the nuclear mask and enlarge it several times to capture the cytoplasmic or membrane signal outside the nucleus. This approach has an advantage of preserving a uniform relationship between nuclear mask and cytoplasmic mask under most circumstances. Mean fluorescence intensity of Lrp8 were calculated for each section by dividing total fluorescence intensity by number of pixels measured for each soma. The intensity of synaptic proteins in neuropile was quantified using ImageJ/FIJI. After background subtraction, and the mean signal intensity across the whole image was measured for each protein.

Axon regeneration assay

Healthy uninjured mouse cortical neurons (7 DIV) and iPSC derived human neurons (45-50 DIV) were injured using a multipin scrapping tool and allowed to regenerate for another 7 d before fixation with 4% PFA in PBS. Axon regeneration in the scrape zone were visualized using anti-ßIII tubulin mAb (1:5000, G7121 Promega), phalloidin conjugates for actin staining (1;1000, A12379 and A12381, Invitrogen) and DAPI (1:5000, Bio-Rad). For scoring axon regeneration, images acquired at 10x magnification in an automated high-throughput imager (ImageXpress Micro confocal, Molecular Devices) under identical conditions for all experiments. Image thresholding and quantification were automated using ImageJ script angiotube formation algorithm to analyse axon regrowth(*59*). The entire dataset was manually blinded by the investigator before analysis.

bioRxiv preprint doi: https://doi.org/10.1101/2025.10.10.681747; this version posted October 13, 2025. The copyright holder for this preprint (which was not certified by peer review) is the author/funder, who has granted bioRxiv a license to display the preprint in perpetuity. It is made available under aCC-BY 4.0 International license.

Surface biotinylation and LRP8 recycling

Primary mouse cortical neurons were treated with purified secreted ApoE (12 µg/ml), 24 h after axotomy on day 7 of culture. On day 12 , primary neurons were treated with Reelin (2 µg/ml) for 1 h before performing surface biotinylation assay (see timeline in Fig. 4C). After reelin treatment, the cells were washed with ice cold PBS and incubated in PBS containing sulfo-NHS-SS-Biotin reagent (1.0 µg/ml) for 30 min at 4°C. Rinsing the neurons again with ice cold PBS with 100 mM glycine helps to quench the excess biotin reagent. Neurons were then lysed using 10X RIPA lysis buffer (EMD Millipore 20-188) with EDTA-free protease and phosphatase inhibitors cocktail tablets (Roche 11873580001 and 04906837001) at 4°C for 20 min. Debris was removed by centrifugation for 15 min at 15,000 rpm at 4°C. Protein estimated using PierceTM BCA protein assay kit (#23225). 100 µg of total protein was incubated with 50 µl of NeutrAvidin agarose pellets at 4°C for 1 hr. Biotinylated surface proteins that are bound to be agarose pellets was washed thrice in washing buffer containing 500 mM NaCl, 15 mM Tris-HCl, 0.5% Triton X-100 at pH 8.0. Biotinylated surface proteins eluted from beads by boiling in 4X SDS sample loading buffer and analyzed on SDS-PAGE. The following antibodies were used for immunoblot: anti-ApoE (1:1000 ab52607), anti-LRP8 (1:500 4H3E6 MABN1872), anti-LRP5 (1:1000 D80F2 #5731), anti-LRP6 (1:500 C5C7 #2560), anti-Src (1:1000 36D10), anti-phospho-Src family (Tyr416, D49G4 1:1000). Immunoblots developed using Infrared dyes of 680 and 800 dyes in LI-COR Odyssey imaging systems. Single channel, high-resolution tiff images were converted to grey scale for densiometric analysis using Image Studio Lite image processing software.

Single nuclei 10x genomic sequencing and analysis

Mouse brain tissue collection and nuclei isolation for single nuclei RNA-seq were completed as previously described with slight modification(*60*). E2 and E4 mice were aged for 6 weeks and then underwent either a sham or dorsal thoracic over-hemisection surgical procedure. Eleven weeks post-surgery, E2 and E4 mice with and without SCI were sacrificed via rapid dissection. For each mouse, the motor cortex and corresponding sub-cortical regions from the right-brain hemisphere were micro-dissected, pooled, and immediately frozen on dry ice then stored at -80°C until nuclei isolation could be performed. Individual tissues were separately homogenized and then underwent density-based nuclei separation via centrifuging for one hour. Nuclei pellets were obtained, resuspended, and counted on a hemocytometer to achieve a concentration of 700-1200 nuclei/µl for generating single nuclei cDNA libraries.

Barcode-incorporated single-nucleus cDNA libraries were constructed using the Chromium Single Cell 3' Reagents Kit v3 (10x Genomics) following the manufacturer's guidelines. Sample cDNA libraries of all experimental groups were pooled and batched sequenced on an Illumina NovaSeq 5000 using single-indexed paired-end HiSeq sequencing. A sequencing depth of >400 million reads was achieved for all samples with an average read depth of 50,000 reads per nuclei. The resulting sequencing binary base call files (BCLs) were demultiplexed into FASTQ files. Sequenced samples were aligned to the mm10-2020-A *Mus musculus* reference genome using the Cell Ranger Count software (pipeline version 7.1, 10x Genomics), generating barcoded sparse matrices of nuclei-gene raw UMI counts.

Sample nuclei-gene count matrices were imported and combined into a single anndata object for quality control (QC) and downstream processing with Scanpy (version 1.9.3)(*61*), a scalable toolkit for Python-based gene expression analyses. Individual samples with total nuclei counts outside the interquartile range (IQR) of the data set were removed to improve the reliability of the results. Genes detected in less than 10 nuclei were discarded. Nuclei with over 5% of UMI counts mapped to mitochondria genes and nuclei with less than 50 genes or more than 5,000 genes detected were considered outliers and discarded. Raw nuclei-gene counts were retained as a separate layer within the annotated object for integrated clustering. For comparative gene expression analysis, the UMI counts were normalized to their library size by scaling to 10,000 transcripts per nucleus and then log-transformed. Across all experimental groups, 201,941 nuclei and 26,473 genes were retained post-QC processing and clustering.

In total, 20 samples were used for integrated clustering (E4, n=5; E2, n=4; E4-SCI, n=5; E2-SCI, n=6). Sample clustering was performed by first using Scanpy's *highly_variable_genes* (HVGs) function, and the implementation of *'flavor=seurat_v3'*, to identify shared HVGs between samples(*62, 63*). Briefly, this method computes a standardized normalized variance by which genes are ranked. Genes are then sorted by their variance and rank-matched between samples, with the top 2500 genes selected as HVGs based on the number

bioRxiv preprint doi: https://doi.org/10.1101/2025.10.10.681747; this version posted October 13, 2025. The copyright holder for this preprint (which was not certified by peer review) is the author/funder, who has granted bioRxiv a license to display the preprint in perpetuity. It is made available under aCC-BY 4.0 International license.

of samples with shared rankings. Next, the scVI probabilistic model from the scVI-tools package was used for sample-based batch correction, incorporating covariate regression of total library size and mitochondrial transcripts per nucleus(*64*). The model was then trained to obtain the latent space representation of each nucleus.

A neighborhood graph of the batch-corrected matrix was constructed using Scanpy's *neighbourhoods* function, with *'n_neighbourhoods'* size of 15 and the scVI-generated latent space representation as input parameters. Integrated Leiden clustering of nuclei into cell-type subgroups was performed from the neighbourhood graph and dimensionally reduced in UMAP space for visualization. To enhance the clustering process, nuclei identified as doublets were excluded from the dataset, and the integrated Leiden clustering was repeated using a newly retrained scVI model. Doublets were identified based on clusters showing enrichment for marker genes of different cell types, suggesting mixed-cell expression profiles within the same cluster.

Clusters of nuclei enriched with specific marker genes were classified as corresponding primary cell types. To identify the top differentially expressed marker genes within individual Leiden clusters, the *rank_genes_groups* function in Scanpy was used with the Wilcoxon Ranked-Sum test. Genes with the highest ranks showing unique enrichment in a single cluster were considered marker genes. The cell type specificity of these markers was verified using previously published data(*60, 65, 66*).

Additionally, the *rank_genes_groups* function with the Wilcoxon Ranked-Sum test was employed for differential gene expression (DEG) analysis between experimental groups for each major identified cell type. Some sub-clusters, enriched with nuclei from a single sample, were excluded from all DEG analyses. Using the remaining clusters, only genes expressed in at least 10% of all nuclei post-ranking were considered differentially expressed. Significant DEGs were defined as those with an adjusted *p-value* (false discovery rate) of less than 0.05 and a Log_2 fold-change greater than ±0.25, unless specified otherwise (Table S1).

Gene set enrichment analysis of DEGs from select cell types was conducted using Cytoscape (version 3.9.1)(*67*) with ClueGo (version 3.10.2)(*68*) extensions. DEG lists were analyzed in ClueGo to identify overrepresented functional and pathway terms using the Gene Ontology (GO) Biological Processes, Molecular Functions, and Reactome Pathways and Reactions databases. A two-sided hypergeometric test with BH-adjusted p-value of 0.005 and a 5-gene threshold was used for term enrichment. GO term fusion was applied to merge redundant and related biological themes with more than 50% gene similarity. Filtered term associations were visually organized into functionally related clustered network using the GeneMANIA-based clustering algorithm(*69, 70*). Selected GO-term associated gene subsets were further assessed for protein-protein interactions (PPI) using STRING (version 12)(*71, 72*), visualizing PPI networks with a confidence score of 0.3 for gene association strength.

Statistics
Statistical analysis was performed in Prism 10.3.1. All data presented here as mean $\pm$ SEM unless specified otherwise. Two-way ANOVA with repeated measures was performed SPSS for time course datasets of repeated measurements. Student's two-tailed unpaired t-test and one-way ANOVA with Tukey's multiple comparisons test were used for pairwise comparisons. Statistical significance was set as $p < 0.05$ for all analysis. The specific test used to calculate *p* value, number of replicates, F and Df are noted in Fig. legends.

bioRxiv preprint doi: https://doi.org/10.1101/2025.10.10.681747; this version posted October 13, 2025. The copyright holder for this preprint (which was not certified by peer review) is the author/funder, who has granted bioRxiv a license to display the preprint in perpetuity. It is made available under aCC-BY 4.0 International license.

SUPPLEMENTARY FIGURES S1-S7

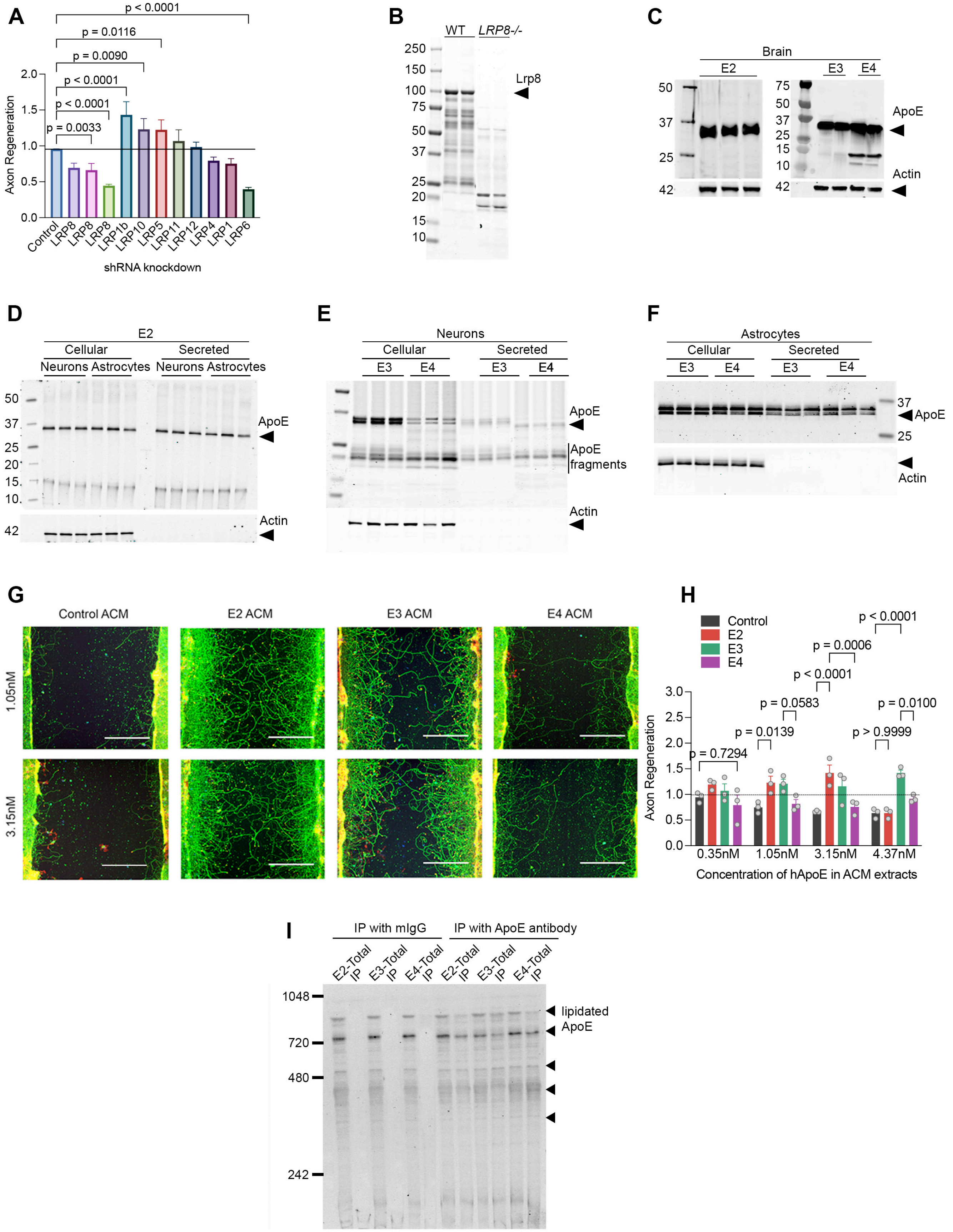

bioRxiv preprint doi: https://doi.org/10.1101/2025.10.10.681747; this version posted October 13, 2025. The copyright holder for this preprint (which was not certified by peer review) is the author/funder, who has granted bioRxiv a license to display the preprint in perpetuity. It is made available under aCC-BY 4.0 International license.

Supplementary Fig. S1. Role of ApoE-Lrp8 in axon regeneration.

(A) Quantification of axon regeneration in WT cortical neurons cultured with shRNA-mediated knockdown for different members of LDL receptor-related protein (LRP) family. Data represents mean + SEM from three biological replicates and analyzed with one-ANOVA with Dunnett's multiple comparison test (F = 6.25, dF = 11).
(B) Immunoblot of RIPA total protein extracts isolated from brain of WT and *Lrp8*$^{-/-}$ to access the specificity of LRP8 antibody.
(C) Anti-ApoE immunoblot of RIPA extracted total protein isolated from mouse brain carrying the indicated human APOE-KI alleles. Anti-actin used as loading control.
(D) Anti-ApoE immunoblot analysis of RIPA extracts from cellular and secreted fractions of cortical neuronal and astrocytes cultures from E2 mice. Anti-actin used as loading control.
(E) Anti-ApoE immunoblot analysis of RIPA extracts from cellular and secreted fractions of cortical neuronal cultures from E3 and E4 mice. Anti-actin used as loading control.
(F) Anti-ApoE immunoblot analysis of RIPA extracts from cellular and secreted fractions of cortical astrocytes cultures from E3 and E4 mice. Anti-actin used as loading control.
(G) Photomicrographs of axon regeneration in cultured cortical neurons from EKO mice treated with astrocyte conditioned medium (ACM) containing 1.05 nM and 3.15 nM of hApoE derived from cortical astrocytic cultures from respective APOE-KI mice. Cultures stained at d15 for ßIII-tubulin (green) and phalloidin (red). Scale bar, 200 µm.
(H) Quantification of axon regeneration from (G). Data shown as mean + SEM from three biological replicates and analyzed by two-way ANOVA with Tukey's multiple comparisons test with F (9, 32) = 4.5, p = 0.0007 for interaction effects, F (3. 32) = 18.45, p<0.0001 for different ACM effects and F (3, 32) = 0.86, p = 0.46 between different ACM concentrations.
(I) Native PAGE analysis to detect ApoE lipoprotein profile after immunoprecipitation with mIgG and anti-hApoE using ACM extracts derived from astrocytic cultures of hAPOE-KI mice.

bioRxiv preprint doi: https://doi.org/10.1101/2025.10.10.681747; this version posted October 13, 2025. The copyright holder for this preprint (which was not certified by peer review) is the author/funder, who has granted bioRxiv a license to display the preprint in perpetuity. It is made available under aCC-BY 4.0 International license.

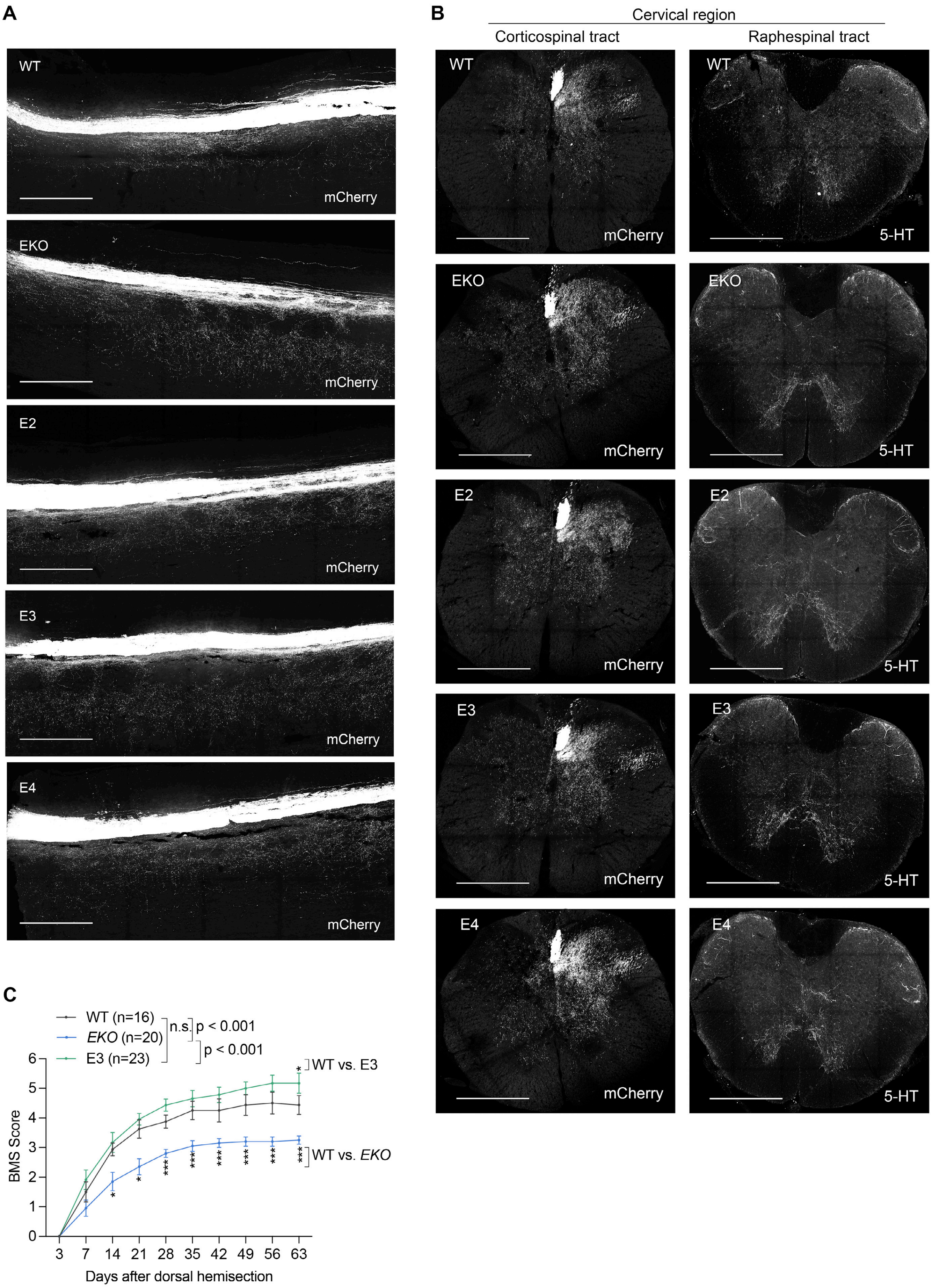

bioRxiv preprint doi: https://doi.org/10.1101/2025.10.10.681747; this version posted October 13, 2025. The copyright holder for this preprint (which was not certified by peer review) is the author/funder, who has granted bioRxiv a license to display the preprint in perpetuity. It is made available under aCC-BY 4.0 International license.

Supplementary Fig. S2. Development of corticospinal and raphespinal tracts in hAPOE-KI mice and its role in SCI recovery.

(A) Sagittal photomicrographs of thoracic region of spinal cord. WT (*n* = 3), EKO (*n* = 4), E2 (*n* = 3), E3 (*n* = 3), E4 (*n* = 3) mice, injected into M1 cortex with AAV-based anatomical tracer to label CST axon bundle. Sections stained with anti-mCherry to access CST development. Dorsal is up and rostral is left. Scale bar, 200 μm.

(B) Transverse photomicrographs of cervical region of spinal cord stained with anti-mCherry to access CST tract and with anti-5HT to access development of raphespinal tract in the experimental cohort described in Fig. 2A. Scale bar, 200 μm.

(C) Open-field performance score for locomotion by BMS score of WT (*n* = 16), EKO (*n* = 20) and E3 (*n* = 23). Performance scores were recorded for each animal every consecutive week and data reported as mean ± SEM and analyzed using repeated measure ANOVA across time series followed by post hoc Tukey's multiple comparisons test for genotypic effect at indicated time points. ***$p<0.001$, *$p<0.05$.

bioRxiv preprint doi: https://doi.org/10.1101/2025.10.10.681747; this version posted October 13, 2025. The copyright holder for this preprint (which was not certified by peer review) is the author/funder, who has granted bioRxiv a license to display the preprint in perpetuity. It is made available under aCC-BY 4.0 International license.

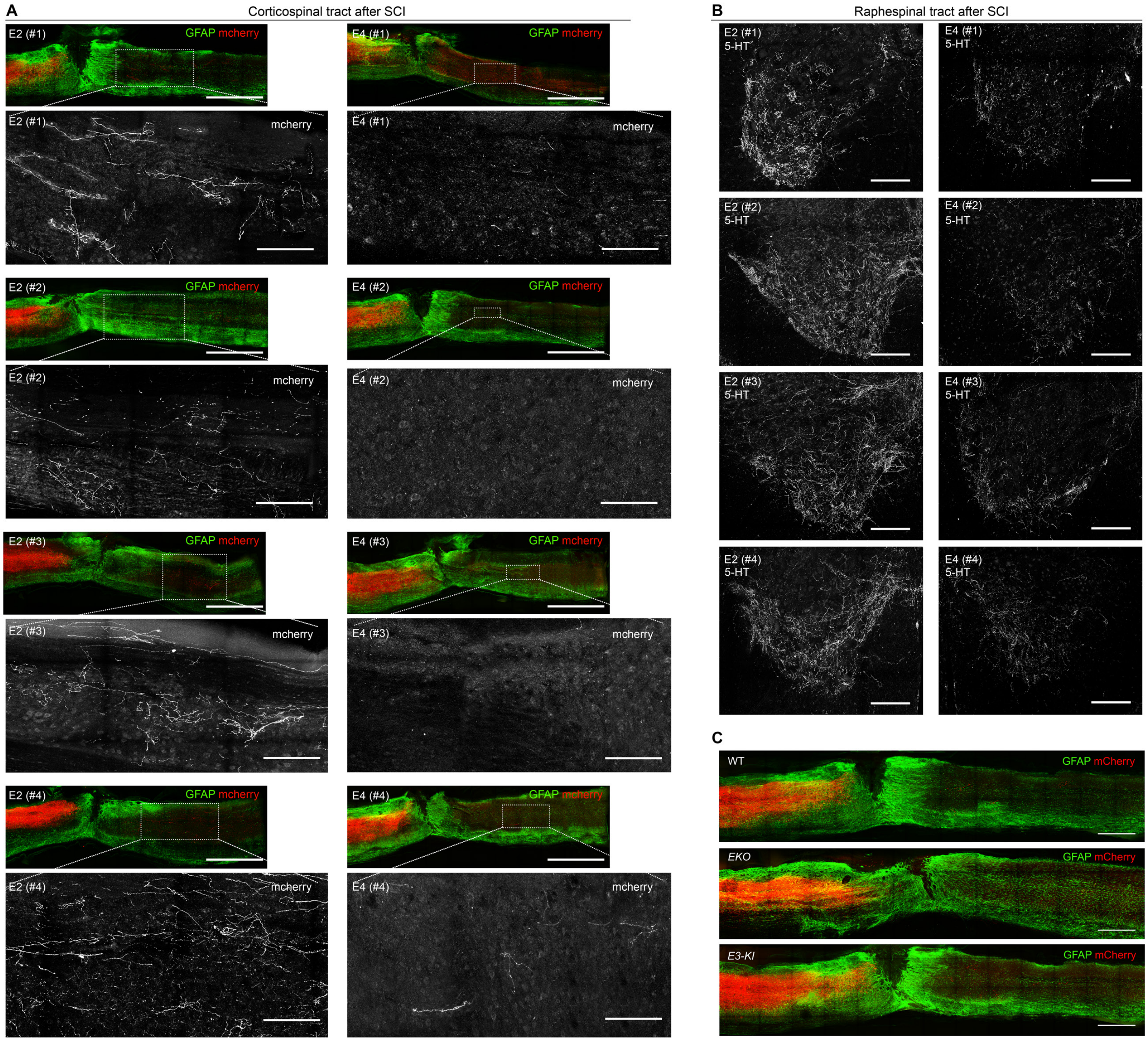

bioRxiv preprint doi: https://doi.org/10.1101/2025.10.10.681747; this version posted October 13, 2025. The copyright holder for this preprint (which was not certified by peer review) is the author/funder, who has granted bioRxiv a license to display the preprint in perpetuity. It is made available under aCC-BY 4.0 International license.

Supplementary Fig. S3. E2 mice exhibit pronounced reparative axon regrowth after SCI.

(A) Sagittal low power photomicrographs of spinal cord around the lesion site in E2 and E4 mice. Sections were stained with anti-GFAP (green) and anti-mCherry (red). Dorsal is up and rostral is left. Scale bar, 500 µm. White outlined boxed areas in each image are captured at high resolution to visualize regenerating CST fibers caudal to lesion for red channel only. Scale bar, 100 µm.

(B) Transverse section photomicrograph of ventral horn lumbar spinal cord of E2 and E4 mice. Sections stained with anti-5-HT to visualize raphespinal fibers. Scale bar, 100 µm.

(C) Sagittal photomicrographs of spinal cord around the lesion site in WT (*n* = 12), *EKO* (*n* = 13) and E3 (*n* = 16) mice. Sections were stained with anti-GFAP (green) and anti-mCherry (red). Dorsal is up and rostral is left. Scale bar, 500 µm.

bioRxiv preprint doi: https://doi.org/10.1101/2025.10.10.681747; this version posted October 13, 2025. The copyright holder for this preprint (which was not certified by peer review) is the author/funder, who has granted bioRxiv a license to display the preprint in perpetuity. It is made available under aCC-BY 4.0 International license.

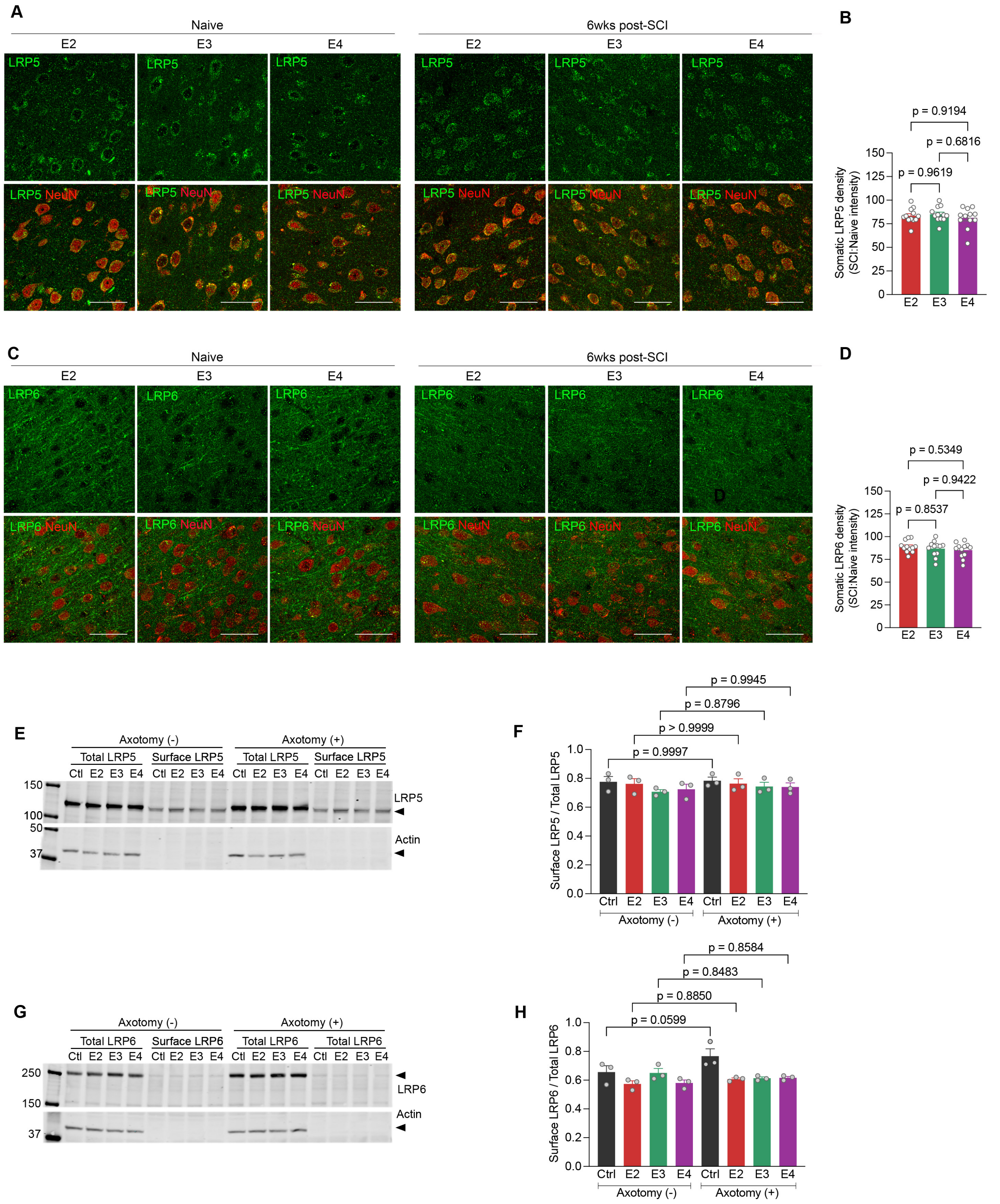

bioRxiv preprint doi: https://doi.org/10.1101/2025.10.10.681747; this version posted October 13, 2025. The copyright holder for this preprint (which was not certified by peer review) is the author/funder, who has granted bioRxiv a license to display the preprint in perpetuity. It is made available under aCC-BY 4.0 International license.

Supplementary Fig. S4. LRP5 and LRP6 receptors not required for ApoE dependent reparative axon growth.

(A) Photomicrographs of L5 from M1 brain cortex stained for anti-LRP5 (green) and anti-NeuN (red) 42 days after SCI compared to age-matched naïve controls for respective genotypes. Scale bar, 200 µm.
(B) Quantification of LRP5 levels in neuronal soma 42 days after SCI for E2, E3 and E4 mice (*n* = 12). Data presented as mean ± SEM and analyzed two-tailed unpaired t test (t = 0.54, dF = 22).
(C) Photomicrographs of L5 from M1 brain cortex stained for anti-LRP6 (green) and anti-NeuN (red) 42 days after SCI compared to age-matched naïve controls for respective genotypes. Scale bar, 200 µm.
(D) Quantification of LRP6 levels in neuronal soma 42 days after SCI for E2, E3, E4 mice (*n* = 12). Data presented as mean ± SEM and analyzed two-tailed unpaired t test (t = 1.29, dF = 22).
(E) Immunoblot with total and biotinylated surface protein extracts from Fig. 4J to measure differential recycling of surface LRP5 with actin as loading control.
(F) Densitometry analysis of immunoblots from three independent experiments described in Fig. 4J for surface LRP5 with excess reelin. Data presented as mean ± SEM and analyzed by one-way ANOVA with Tukey's multiple comparisons test with F = 0.73, dF = 7.
(G) Immunoblot with total and biotinylated surface protein extracts from (Fig. 4J) to measure differential recycling of surface LRP6 with actin as loading control.
(H) Densitometry analysis of immunoblots from three independent experiments described in (Fig. 4J) for surface LRP6 with excess reelin. Data presented as mean ± SEM and analyzed by one-way ANOVA with Tukey's multiple comparisons test with F = 4.53, dF = 7.

bioRxiv preprint doi: https://doi.org/10.1101/2025.10.10.681747; this version posted October 13, 2025. The copyright holder for this preprint (which was not certified by peer review) is the author/funder, who has granted bioRxiv a license to display the preprint in perpetuity. It is made available under aCC-BY 4.0 International license.

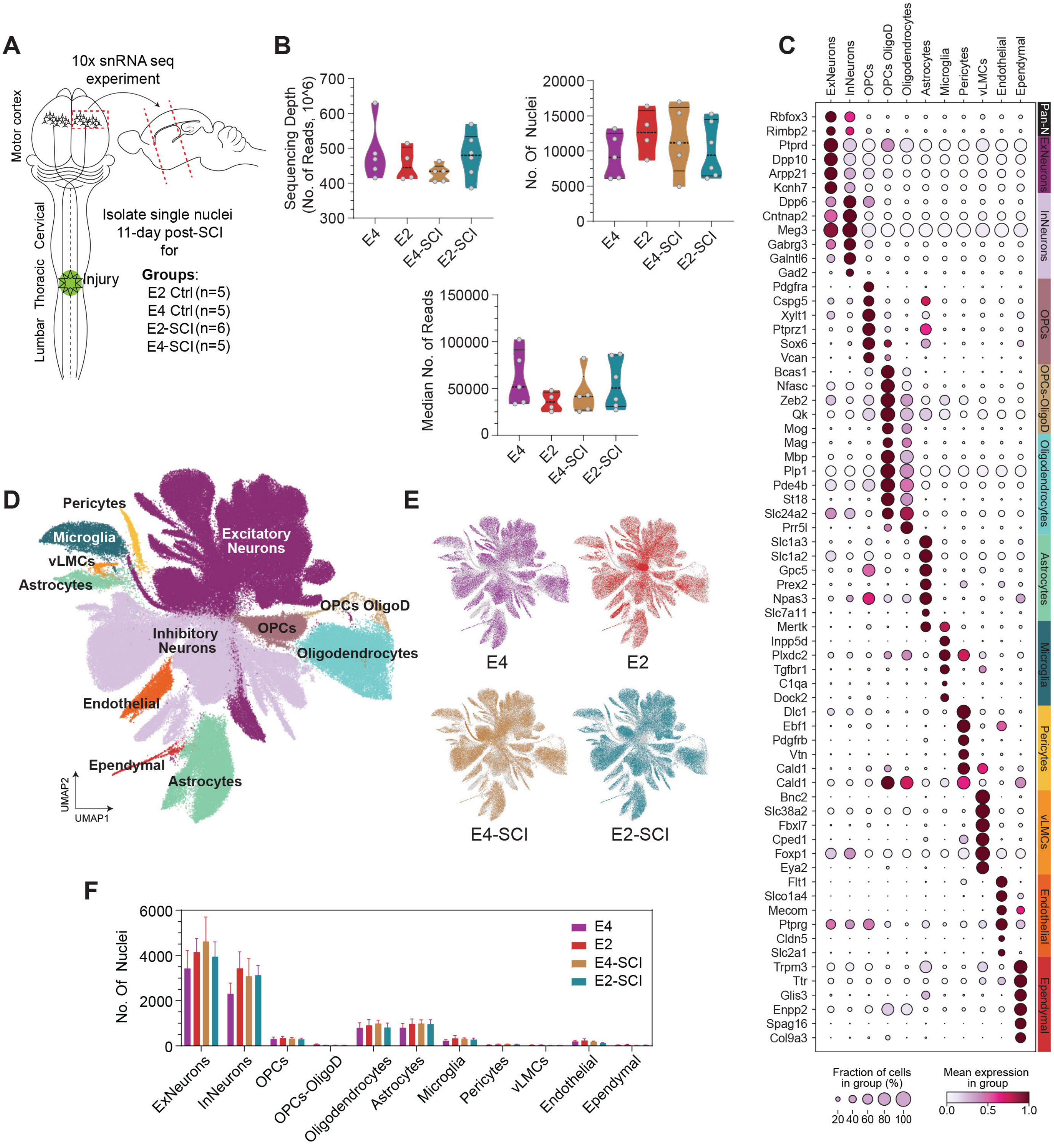

bioRxiv preprint doi: https://doi.org/10.1101/2025.10.10.681747; this version posted October 13, 2025. The copyright holder for this preprint (which was not certified by peer review) is the author/funder, who has granted bioRxiv a license to display the preprint in perpetuity. It is made available under aCC-BY 4.0 International license.

Supplementary Fig. S5. Integration of snRNA-seq datasets and classification of major cell types.

(A) Schematic depicting the overview of experimental design, brain region used for single nuclei isolation and RNA sequencing across E2 and E4 experimental mice with pre- and post-injury.
(B) The sequencing depth (left), number of total nuclei (right), and median number of reads (bottom) resulting from the single-nucleus RNA-sequencing (snRNAseq) of samples pooled by experimental groups.
(C) UMAP of cell-type identified clusters.
(D) Dot plot showing the percentage of nuclei and scaled mean expression of cell-type specific marker genes used to determine the UMAP clusters in C.
(E) UMAP of collective sample gene expression profiles projections for each experimental group.
(F) Comparative nuclei frequencies of clustered cell types of individual samples within each experimental group. Data is presented as the mean ± SEM and compared by a mixed model test with Tukey's correction. No between-group statistical significance differences were detected.

bioRxiv preprint doi: https://doi.org/10.1101/2025.10.10.681747; this version posted October 13, 2025. The copyright holder for this preprint (which was not certified by peer review) is the author/funder, who has granted bioRxiv a license to display the preprint in perpetuity. It is made available under aCC-BY 4.0 International license.

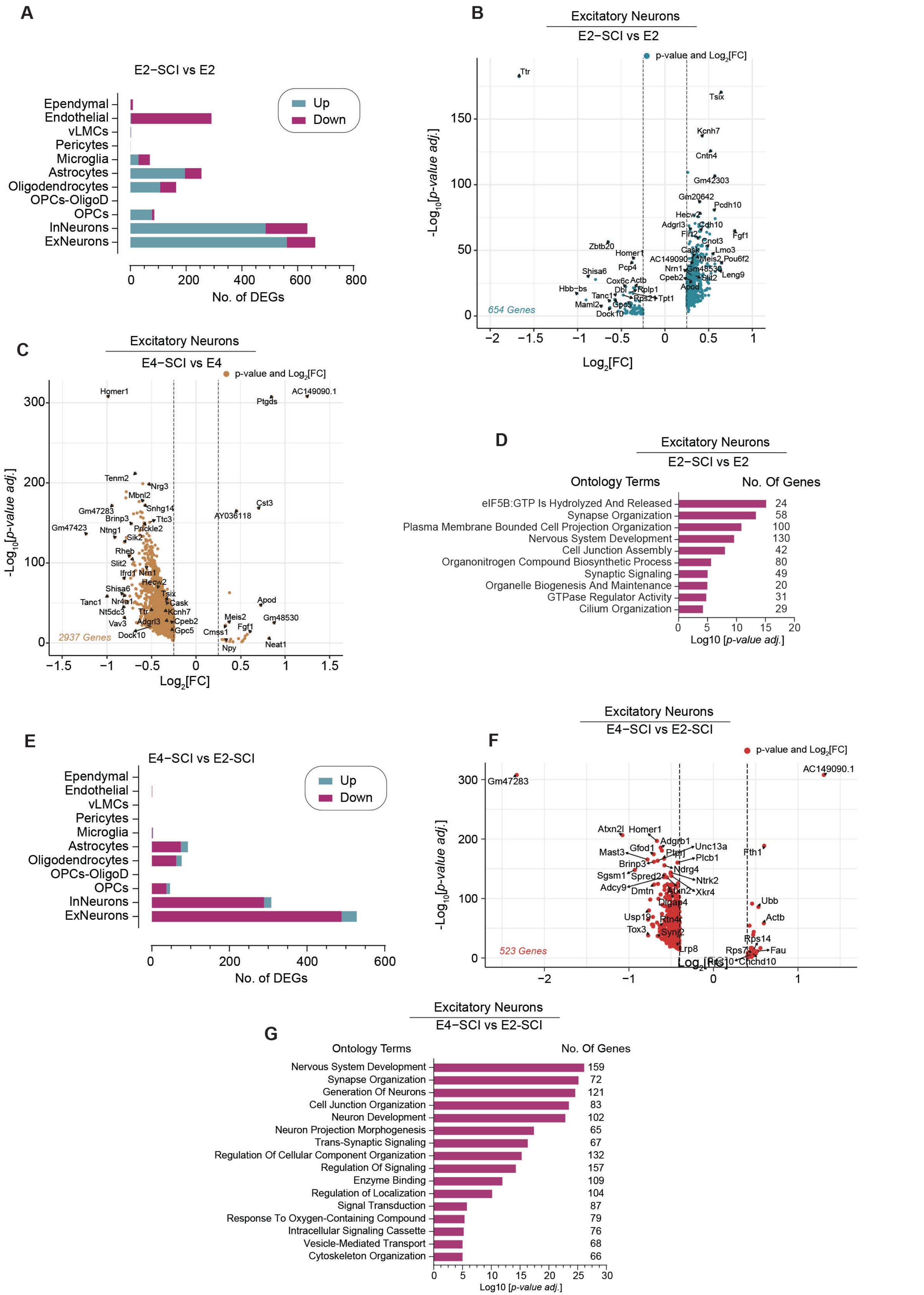

bioRxiv preprint doi: https://doi.org/10.1101/2025.10.10.681747; this version posted October 13, 2025. The copyright holder for this preprint (which was not certified by peer review) is the author/funder, who has granted bioRxiv a license to display the preprint in perpetuity. It is made available under aCC-BY 4.0 International license.

Supplementary Fig. S6. Cortical upregulation of SCI-induced growth-promoting transcriptomic signature is specific to the E2 allele.

(A) The number of up and downregulated differentially expressed genes (DEGs) in neuronal and glial cell populations in E2 mice post-SCI.
(B) Volcano plots representing the gene expression changes in the excitatory neuron populations of E2 mice post-SCI.
(C) Volcano plots representing the gene expression changes in the excitatory neuron populations of E4 mice post-SCI.
(D) Bar plot of the *p*-values, and corresponding number of term-associated genes, for the top leading terms from gene set pathway enrichment analysis of E2 excitatory neurons DEGs post-SCI shown in A. Complete list of enriched pathway terms can be found in Supplementary Table S3.
(E) Number of up and down regulated DEGs in neuronal and glial cell populations in RAG category.
(F) Volcano plot showing gene expression changes in excitatory neuron cells in RAG category.
(G) Bar plot of the *p*-values, and corresponding number of term-associated genes, for the top leading terms from gene set pathway enrichment analysis of E2 excitatory neurons DEGs post-SCI shown in E. Complete list of enriched pathway terms can be found in Supplementary Table S4.

bioRxiv preprint doi: https://doi.org/10.1101/2025.10.10.681747; this version posted October 13, 2025. The copyright holder for this preprint (which was not certified by peer review) is the author/funder, who has granted bioRxiv a license to display the preprint in perpetuity. It is made available under aCC-BY 4.0 International license.

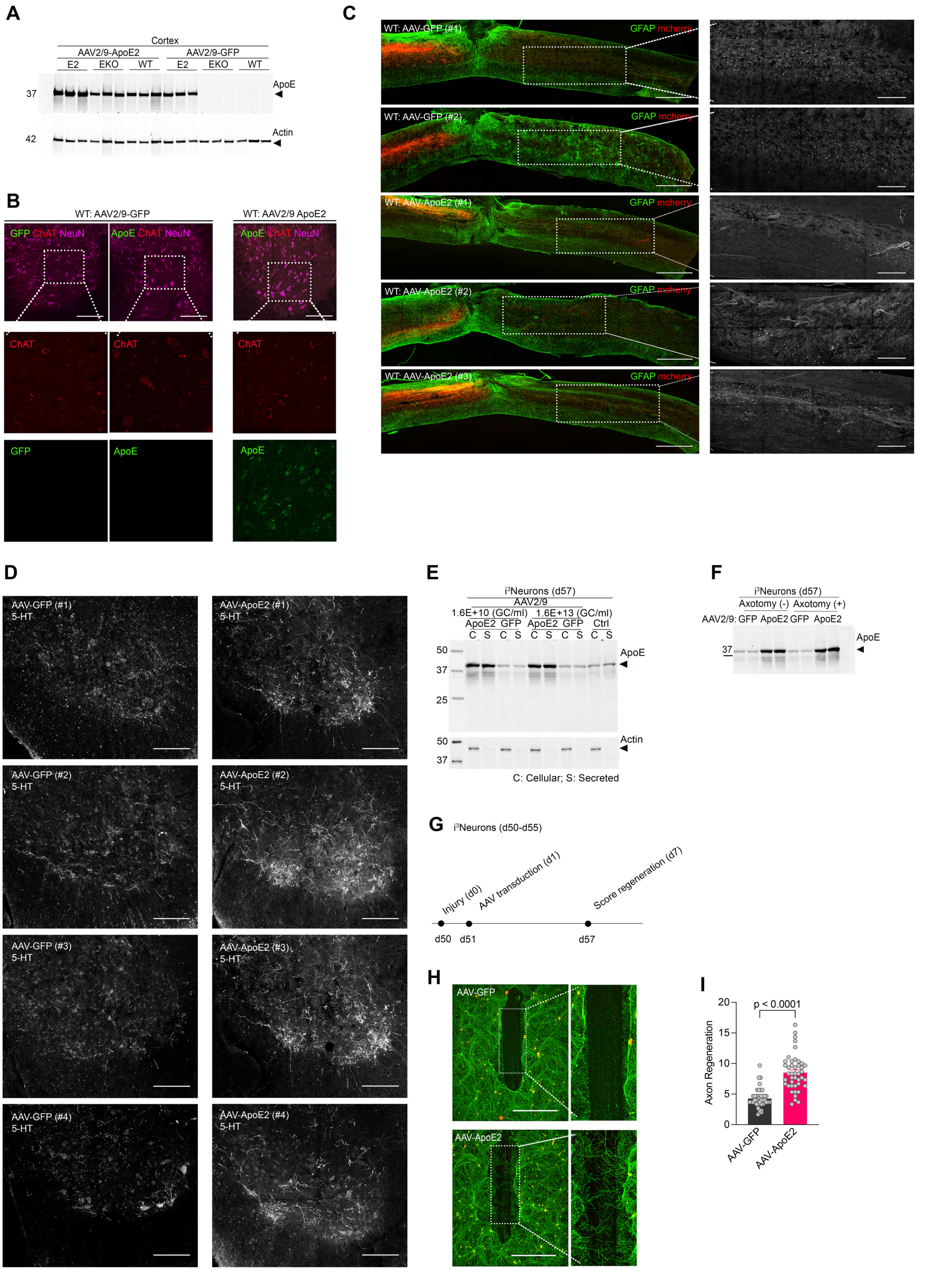

bioRxiv preprint doi: https://doi.org/10.1101/2025.10.10.681747; this version posted October 13, 2025. The copyright holder for this preprint (which was not certified by peer review) is the author/funder, who has granted bioRxiv a license to display the preprint in perpetuity. It is made available under aCC-BY 4.0 International license.

Supplementary Fig. S7. Expression profile analysis of AAV-mediated over expression of ApoE2 in cortex and i3N-derived human neurons.

(A) Anti-ApoE immunoblot with total protein extracts from forebrain of WT, EKO and E2 mice with and without bilateral AAV2/9 injection at M1 cortex. Anti-actin used for loading control.

(B) Transverse section photomicrograph of ventral horn lumbar spinal cord of WT mice at d77 (related to Fig. 7). Sections stained with anti-GFP, ApoE, ChAT and NeuN. Scale bar, 100 μm.

(C) Sagittal low-power photomicrographs of spinal cord around the lesion site in AAV injected WT mice at d77 after SCI. Sections were stained with anti-GFAP (green) and anti-mCherry (red). Dorsal is up and rostral is left. Scale bar, 500 μm. White outlined boxed areas in each image are captured at high-resolution to visualize regenerating CST fibers caudal to lesion for red channel only. Scale bar 100 μm.

(D) Transverse section photomicrograph of ventral horn lumbar spinal cord of WT mice expressing GFP and ApoE2. Sections stained with anti-5-HT. Scale bar, 100 μm.

(E) Anti-ApoE immunoblot profile with cellular and secreted extracts of AAV2/9 transduced i3N neurons at d57. GFP expression is used as control to access levels of expressed ApoE2 compared to endogenous ApoE. Anti-actin used as loading control.

(F) Anti-ApoE immunoblot profile with cellular extracts of AAV-GFP and ApoE2 transduced i3N neurons with and without axotomy.

(G) Schematic to evaluate therapeutic benefit of ApoE2 in i^3N-derived human neurons.

(H) Photomicrograph showing regeneration zone of axotomized i^3Neurons stained for ßIII-tubulin (green) and phalloidin (red). White outline in the axon regeneration zone is enlarged.

(I) Quantification of axon regeneration index for i^3N neurons expressing AAV-GFP and AAV-ApoE2 expression. Datapoint refers to each well from three independent replicates performed with different i^3N-iPSC clones. *p* values calculated by two-tailed unpaired t-test with Welch's correction (t = 8.35, dF = 64).
